# Geographic Influence and Metabolomics-Driven Discovery of 5-Alpha Reductase Inhibitors in *Tectona grandis* L.f. (Teak) Leaves

**DOI:** 10.3390/molecules30142895

**Published:** 2025-07-08

**Authors:** Nutchaninad Tanuphol, Corine Girard, Prapapan Temkitthawon, Nungruthai Suphrom, Nitra Nuengchamnong, Tongchai Saesong, Kamonlak Insumrong, Abdulaziz Wadeng, Wiyada Khangkhachit, Andy Zedet, Ratchadaree Intayot, Siriporn Jungsuttiwong, Anuchit Plubrukarn, Francois Senejoux, Kornkanok Ingkaninan

**Affiliations:** 1 Center of Excellence for Natural Health Product Innovation and Center of Excellence for Innovation in Chemistry, Department of Pharmaceutical Chemistry and Pharmacognosy, Faculty of Pharmaceutical Sciences, Naresuan University, Phitsanulok 65000, Thailand; nutchaninad789@gmail.com (N.T.); prapapantem@gmail.com (P.T.); tongchai_saesong@hotmail.com (T.S.); khangkhachit.w@outlook.com (W.K.); 2 Université Marie et Louis Pasteur, EFS, INSERM RIGHT (UMR1098), F-25000 Besançon, France; corine.girard@univ-fcomte.fr (C.G.); andy.zedet@univ-fcomte.fr (A.Z.); francois.senejoux@univ-fcomte.fr (F.S.); 3 Department of Chemistry, Faculty of Sciences, Naresuan University, Phitsanulok 65000, Thailand; suphrom.n1@gmail.com (N.S.); insumrong.k@gmail.com (K.I.); 4 Science Laboratory Center, Faculty of Science, Naresuan University, Phitsanulok 65000, Thailand; nitran@nu.ac.th; 5 Department of Pharmacognosy and Pharmaceutical Botany, Faculty of Pharmaceutical Sciences, Prince of Songkla University, Hat-Yai, Songkhla 90112, Thailand; phoogun001@gmail.com (A.W.); anuchit.pl@psu.ac.th (A.P.); 6 Center of Excellence for Innovation in Chemistry, Department of Chemistry, Faculty of Science, Ubon Ratchathani University, Ubon Ratchathani 34190, Thailand; ratchadaree.in.64@ubu.ac.th (R.I.); siriporn.j@ubu.ac.th (S.J.)

**Keywords:** *Tectona grandis* L.f., steroid 5-alpha reductase inhibitor, metabolomics, structure elucidation

## Abstract

The inhibition of steroid 5-alpha reductase (S5AR), a key mechanism for managing dihydrotestosterone-dependent conditions, has been demonstrated in teak (*Tectona grandis* L.f.) leaf extracts. Our recent clinical study confirmed the effectiveness of a hair growth formulation containing teak leaf extract in males with androgenic alopecia. However, significant variability in S5AR inhibitory activity among teak leaf samples from different regions underscores the need for quality control of raw materials. This study applied a metabolomics approach to investigate the influence of leaf age, harvesting period, and geographic origin on chemical composition and S5AR inhibitory activity, as well as to identify active S5AR inhibitors. Geographic origin emerged as the primary determinant of variations in chemical profiles and S5AR inhibitory activity. Using orthogonal partial least squares analysis, six diterpenoid S5AR inhibitors were identified, including four compounds reported for the first time as S5AR inhibitors: rhinocerotinoic acid, 7-oxo-8-labden-15-oic acid, 8-hydroxy-labd-13-en-15-oic acid, and a novel diterpene, 7-hydroxy-labd-8,13-dien-15-oic acid. These findings highlight the potential of metabolomics as a powerful tool for discovering bioactive compounds and optimizing raw material selection. By prioritizing proven geographic sources, consistent bioactivity can be achieved, supporting the therapeutic potential of teak leaves in managing S5AR-related conditions.

## 1. Introduction

The enzymatic activity of steroid 5 alpha-reductase (S5AR) plays a pivotal role in the conversion of testosterone to dihydrotestosterone (DHT), a potent androgen linked to the pathogenesis of DHT-dependent diseases such as benign prostatic hyperplasia, androgenic alopecia, hirsutism, and acne [1]. The inhibition of this enzyme offers a promising therapeutic strategy for slowing the progression of these conditions [2].

*Tectona grandis* L.f., commonly known as teak, is a perennial tree species renowned for its durable timber and its diverse applications in the furniture and construction industries. Beyond its economic significance, various parts of the teak tree possess a rich chemical composition with potential pharmaceutical activities, offering benefits for medical purposes [3,4]. One particular biological activity of teak leaves is their ability to inhibit S5AR [5]. Recently, teak leaf extract has been used as an active ingredient in a hair growth promotion formulation, and its effectiveness and safety have been demonstrated through a randomized, double-blind, placebo-controlled study involving 81 male subjects with androgenic alopecia [6]. Two diterpenes, (+)-Eperua-8,13-dien-15-oic acid and (+)-Eperua-7,13-dien-15-oic acid, previously isolated from teak leaves, have been validated as S5AR inhibitors and used as quality control markers for the extract [7]. However, preliminary studies revealed significant variation in S5AR inhibitory activity among teak leaf samples harvested from different sources. This problem underscores a critical research question regarding the prevalence and significance of such compounds within the intricate matrix of teak leaves, which might be influenced by seasonal variations, environmental factors, and geographical origin [8]. The complex natural components of plants hold implications for biomarker exploration, which is crucial for maintaining the quality and efficacy of natural products.

Metabolomics, a subset of omics-based research, involves the comprehensive analysis of metabolites within biological systems. It has become a powerful tool in the field of natural product research, allowing researchers to understand how secondary metabolites respond to environmental factors and discover bioactive compounds with pharmacological potential [9]. For instance, metabolomics has been used to examine seasonal variations in *Bacopa* species [10], Tieguanyin tea [11], and *Leucosidea sericea* [12], offering valuable information for quality control in natural product applications.

In this study, we employed a metabolomics approach to investigate the chemical composition of teak leaf extracts, specifically focusing on understanding the factors, including leaf age, seasonal variation, and geographical origin, that influence chemical composition using principal component analysis (PCA). Furthermore, orthogonal partial least square (OPLS) analysis was used to explore the correlation between chemical constituents and biological activity. By combining PCA for exploratory data analysis and OPLS for predictive modeling, we aimed to comprehensively study the chemical components of teak leaf extracts, unravel key relationships among these variables, and facilitate the discovery of novel S5AR inhibitors with therapeutic potential.

## 2. Results and Discussion

### 2.1. Investigation of Steroid 5-Alpha Reductase Inhibitory Activity of Samples

**Figure 1:**
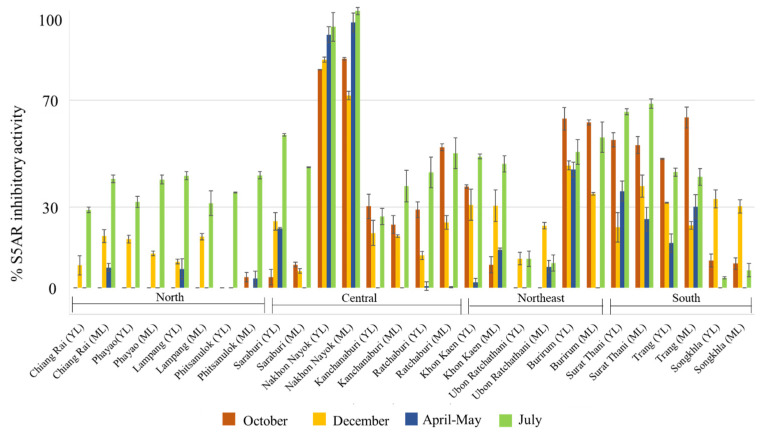
Steroid 5-alpha reductase inhibitory activities of young leaf (YL) and mature leaf (ML) samples collected from 14 provinces in Thailand, classified into four regions: north, central, northeast, and south. The samples were collected in October 2019, December 2019, April–May 2020, and July 2020. The results are presented as the means of triplicate experiments ± standard deviation (SD).

According to typical patterns, teak leaves are shed around December and then bloom again in April, leading to the loss of harvesting samples from Phitsanulok province in December and mature leaves from Saraburi and Burirum provinces in April–May. A total of 108 samples were evaluated for S5AR inhibitory activity. Figure 1 illustrates the significant variation in S5AR inhibitory activity among the teak leaf samples categorized by region. All samples from the north showed a consistent trend of low activity (less than 40% inhibition), while samples from other regions exhibited greater variability. For example, in the central region, the sample from Nakhon Nayok consistently demonstrated promising inhibition of more than 70% across all harvesting periods, while samples from other provinces (Saraburi, Kanchanaburi, Ratchaburi) exhibited low to moderate inhibitory activity. Similarly, samples from the northeast and south also showed low to moderate inhibitory activity (30–70%). These unexpected variations in bioactivity levels prompted further investigation of the effects of leaf age, season, and plant origins on chemical profiles using multivariate analyses.

### 2.2. Investigation of Influence of Different Leaf Ages, Regions, and Harvesting Periods on Chemical Profiles of Teak Leaves Using Principal Component Analysis

**Figure 2:**
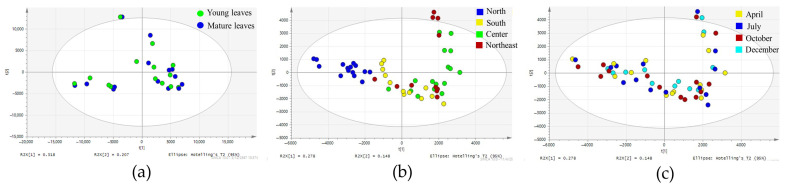
Score plots of PCA. (**a**) Teak leaf samples harvested in October 2019, classified by leaf age. (**b**) Young leaves harvested across all years, classified by regions. (**c**) Young leaves harvested across all years, classified by harvesting periods.

Twenty-eight samples of teak leaves, collected from 14 provinces in October as the initial batch, were analyzed to explore variations in chemical composition between leaf ages. The quality control (QC) samples were tightly clustered at the center of the plot, confirming the reliability of the analytical technique. The PCA results revealed a cumulative R_2_X of 0.80, indicating that 80% of the variance was explained, and a cumulative Q_2_ of 0.52, reflecting moderate but acceptable predictive power across six components. The first principal component, which accounted for the largest variance in the dataset, is shown in Figure 2a. While subsequent components exhibited similar patterns, PCA did not effectively distinguish samples based on leaf age, suggesting that leaf maturity had minimal influence on overall chemical variation. Given this finding, subsequent analyses focused solely on young leaves, which are more consistently available throughout the year.

Figure 2b,c illustrates the PCA results based on the chemical composition of young leaves harvested from 14 sources across four batches: October, December, April–May, and July (55 samples). In these figures, the colors represent plantation regions (Figure 2b) and harvesting periods (Figure 2c), allowing for visual interpretation of grouping trends. The PCA model demonstrated a cumulative R_2_X value of 0.8473 (84.73% of total variance) and a Q_2_ value of 0.63, indicating moderate predictive power. Despite the strong model fit, harvesting time did not significantly influence sample clustering, suggesting that seasonal variation had a limited effect on the chemical profile (Figure 2c). In contrast, the plantation regions had a greater impact, with samples from the northern region forming a distinct cluster separate from other regions (Figure 2b). This trend suggests that geographic origin plays a more prominent role in chemical profile differentiation, while seasonal fluctuations appear to have a negligible effect.

The synthesis of secondary metabolites is influenced by a combination of internal factors, such as genetics, and external factors such as temperature, light, water availability, and insect interactions [8]. Teak leaves contain various groups of secondary metabolites such as flavonoids, phenolics, quinones, coumarins, and terpenes [3,13,14,15]. Our findings align with this complexity, revealing that geographic origin significantly influenced both chemical composition and S5AR inhibitory activity, whereas the effects of leaf age and seasonal variation were less pronounced. Notably, this contrasts with the study of Nayeem and Karveka [15] that reported significant differences in the total phenolic and flavonoid contents between young and mature teak leaves. This discrepancy may be due to the broader scope of our analysis, which examined a full spectrum of secondary metabolites rather than focusing solely on phenolic and flavonoid content. Similarly, while our study found no significant seasonal variations, other research on *Rosmarinus officinalis* L. [16], *Bacopa caroliniana* (Walter) B.L.Rob., and *Bacopa monnieri* (L.) Wettst. [10] has reported substantial seasonal differences in secondary metabolite content. This variation across species may be attributed to species-specific metabolic pathways and differential responses to environmental changes. Although environmental variables such as soil nutrient content and water availability were not recorded in this study, the observed regional differences in chemical profiles strongly suggest that such agro-environmental factors play a contributory role. Future studies integrating controlled cultivation strategies such as soil enrichment and environmental modulation in high-activity regions may help improve raw material consistency and support a comprehensive “farming-to-formulation” approach.

Further studies incorporating longitudinal sampling across multiple years and controlled growth conditions may provide a clearer understanding of the influence of seasonal factors on secondary metabolite production.

### 2.3. The Navigation of Steroid 5 Alpha-Reductase Inhibitors in Teak Leaf Extract Using the Orthogonal Partial Least Squares Model

**Figure 3:**
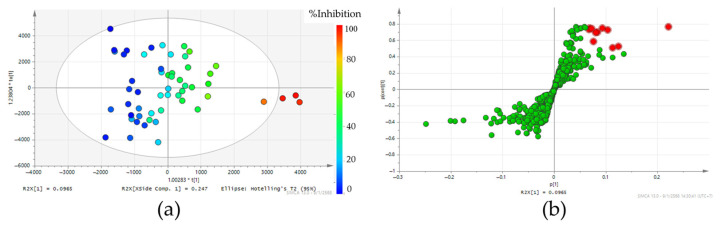
The OPLS plots of teak leaf extracts (young leaves harvested from 14 sources across four batches). (**a**) The score plot represents the correlation between the samples and their S5AR inhibitory activity. The color gradient indicates the levels of S5AR inhibitory activity. (**b**) The S-plot represents the chemical compositions responsible for the discrimination of samples in the score plot. The red highlights indicate the candidate S5AR inhibitors.

The OPLS model is a supervised multivariate statistical analysis method that models the relationship between X (chemical components) and Y (S5AR inhibitory activity) variables. Figure 3a shows the OPLS model score plot, where each point represents one of the 55 young leaf extracts, with colors indicating their respective S5AR inhibitory activity. Samples with high S5AR inhibitory activity are positioned on the right, while those with low activity appear on the left (cumulative R_2_X = 0.91, Q_2_ = 0.70). A total of 763 features were detected, representing the chemical composition of the samples. The transformation of the score plot into a loading plot enabled the identification of the compounds or features responsible for sample discrimination. In this study, the S-plot, a type of loading plot, was used to facilitate the identification of potential S5AR inhibitors. This model provides comprehensive data, with the *x*-axis (p [1]) representing the intensity differences between groups and the *y*-axis (p(corr) [1]) indicating the reliability of correlations. Therefore, features located in the upper-right quadrant of the graph were considered highly reliable and predominantly found in active samples. The candidate S5AR inhibitors were selected based on both p [1] and p(corr) [1] values through a two-step process: First, the top 20 features with the highest p [1] values were selected. Subsequently, only features with a p(corr) [1] value greater than 0.5 were chosen for further analysis (F01–F11), as highlighted in red in Figure 3b.

**Figure 4:**
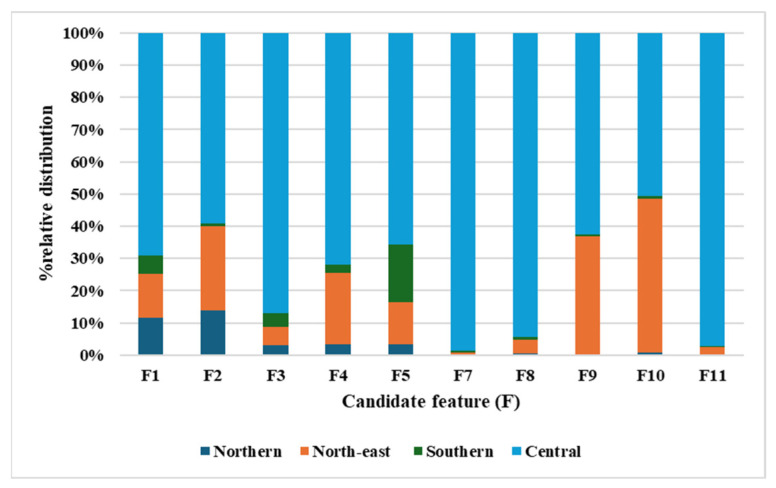
Stacked bar chart representing % relative distribution of candidate features (F1–F11) detected in teak leaf samples across four geographical regions.

Figure 4 presents the stacked bar chart, illustrating the distribution of candidate features (F1–F11) detected in teak leaf samples from four geographical regions of Thailand: northern, central, northeast, and southern. Each feature represents a potential S5AR inhibitor, with the graph showing the relative abundance of these features across the regions.

Notably, samples from the northern region exhibited a consistent trend of lower candidate feature detection compared to other regions. This aligns with the observed S5AR inhibitory activity, where all northern samples demonstrated less than 40% inhibition, suggesting a lower abundance of bioactive compounds contributing to S5AR inhibition. In contrast, the central region displayed a more comprehensive distribution of candidate features across all samples. This distribution aligns with the finding indicating that one sample from the central region (Nakhon Nayok) demonstrated promising S5AR inhibitory activity of over 70% across all harvesting periods, represented by four distinct spots on the right side of the OPLS score plot (Figure 3a). Other samples from the central region (Saraburi, Kanchanaburi, and Ratchaburi) showed varying levels of inhibitory activity, corresponding to moderate detection of candidate features. Similarly, the northeast and southern regions exhibited moderate detection of candidate features, which correlated with their recorded S5AR inhibitory activities ranging from 30% to 70%.

Overall, the graph supports the PCA results, suggesting that geographic origin is the most significant factor influencing chemical composition and its variability in S5AR inhibitory activity across the samples.

### 2.4. Identification of Candidate Compounds

**Figure 5:**
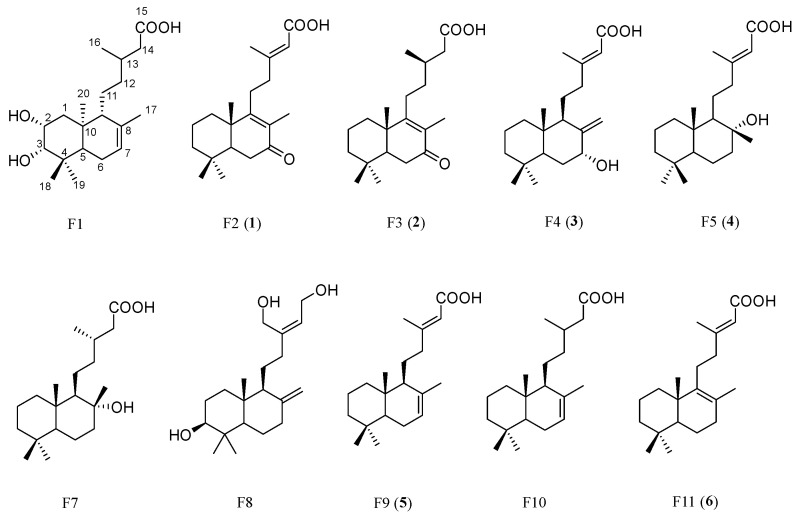
Chemical structures of candidate S5AR inhibitors from teak leaf extract, corresponding to features pinpointed by S-plot analysis of OPLS.

The identification of features F1–F11 was proposed using MS-MS (Table 1 and Table S5). Four compounds (**1**–**4**) corresponding to F2–F5 were isolated and their structures were elucidated and confirmed by NMR and other techniques. The details of this process are discussed in the next section. Two compounds (**5**–**6**) corresponding to F9 and F11 were identified by comparison of their LC retention times and MS-MS data with the isolated compounds from teak leaves obtained in our previous work [7]. The proposed identifications indicated that all the candidate compounds belonged to the diterpene class (Figure 5).

By MS-MS, F1 was tentatively identified as 2,3-dihydroxy-labd-7-en-15-oic-acid, a compound first isolated from *Ophryosporus charrua* (Griseb.) Hieron in 1997 [17]. F6 was confirmed to be a chloride adduct of F7, while F7 was identified as labdanolic acid, which was extracted from the plant *Cistus ladanifer* L. [18]. Labdanolic acid is characterized by a labdane skeleton, which consists of a saturated hydrocarbon framework with a specific arrangement of methyl and hydroxyl groups. This structural feature allows for various chemical transformations, making labdanolic acid a valuable precursor in organic synthesis [19,20]. F8 was identified as tectograndiol, a notable chemical constituent derived from teak. While tectograndiol is known to be present in teak, only a few reviews specifically focused on this compound [21]. F9 and F11 were isomers, identified by MS-MS and comparison with our reference compounds as (+)-eperua-7,13-dien-15-oic acid (**5**) and (+)-eperua-8,13-dien-15-oic acid (**6**), respectively. These compounds were first isolated from *Sindora siamensis* Teijsm. ex Miq. [22] and have also been reported in teak leaves by our group [7]. F10 was identified as labd-7-en-15-oic acid, also known as cativic acid. This compound was initially extracted from the exudate of the cativa tree (*Prioria copaifera* Griseb.) [23] and has more recently been found in *C. ladanifer* [24] and *Ageratina jocotepecana* B.L.Turner [25].

**Table 1:** Mass spectrometry data for 11 candidate features (F1–F11), their proposed identifications, and S5AR inhibitory activities. Curcumin and finasteride are included as reference compounds to provide comparative perspective on potency of teak-derived diterpenoids.

Feature	*m*/*z*	RT (min)	P[1] Value	P(corr)[1] Value	Proposed Molecular Formula	∆ppm	Proposed Compound Identification by MSMS	S5AR Inhibitory Activity (IC_50_, µM)
F1	337.2391	6.83	0.104	0.728	C_20_H_34_O_4_	−1.98	2,3-Dihydroxy-labd-7-en-15-oic-acid [17]	ND
F2	317.2142	7.80	0.076	0.586	C_20_H_30_O_3_	−6.25	Rhinocerotinoic acid (**1**)	54.16 ± 1.73
F3	319.2290	8.14	0.084	0.697	C_20_H_32_O_3_	−3.54	7-Oxo-8-labden-15-oic acid (**2**)	>300 *
F4	319.2290	8.86	0.067	0.732	C_20_H_32_O_3_	−3.54	7-Hydroxy-labd-8,13-dien-15-oic acid (**3**)	21.93 ± 0.67
F5	321.2445	9.47	0.080	0.695	C_20_H_34_O_3_	−3.06	8-Hydroxy-labd-13-en-15-oic acid (**4**)	58.17 ± 0.62
F6	359.2362	10.2	0.093	0.750	C_20_H_36_O_3_ [H + Cl]^−^	0.98	Labdanolic acid (Cl adduct of F.7)	ND
F7	323.2601	10.2	0.227	0.766	C_20_H_36_O_3_	−2.88	Labdanolic acid [19]	ND
F8	321.2449	10.9	0.072	0.744	C_20_H_34_O_3_	−3.06	Tectograndiol [21]	ND
F9	303.2340	15.2	0.114	0.511	C_20_H_32_O_2_	−3.44	(+)-Eperua-7,13-dien-15-oic acid (**5**) [7]	14.65 ± 0.31 [7]
F10	305.2498	15.5	0.125	0.524	C_20_H_34_O_2_	−3.92	Labd-7-en-15-oic acid [24]	ND
F11	303.2341	15.5	0.069	0.748	C_20_H_32_O_2_	−3.45	(+)-Eperua-8,13-dien-15-oic acid (**6**) [7]	14.19 ± 2.87 [7]
Curcumin							92.73 ± 0.14% ** 13.4 ± 0.40 [26] ***	
Finasteride							0.76 ± 0.03 [26] ***	

ND = not determined; * percentage inhibition evaluated at final concentration of 300 µM = 41.41 ± 1.69%; ** percentage inhibition evaluated at final concentration of 50 µg/mL; *** IC_50_ values of curcumin and finasteride are taken from our previous publication [26].

#### Isolation and Structure Elucidation of Compounds **1**–**4**

Chromatographic separation of the crude ethanolic extract focused on the regions containing candidate features, resulting in the successful isolation of four candidate compounds whose structures were then identified by NMR analysis. Three of these were previously identified: F2 was identified as rhinocerotinoic acid (**1**), F3 as 7-oxo-8-labden-15-oic acid (**2**), and F5 as 8-hydroxy-labd-13-en-15-oic acid (**4**). Additionally, a novel compound corresponding to F4 was identified as 7-hydroxy-labd-8,13-dien-15-oic acid (**3**). Among the isolated compounds, compounds **1** and **4** were previously isolated from teak leaf extract [27]. A summary of the compound identification is presented in Table 1.

The isolated compounds (**1**–**4**) belong to the diterpene group that exhibit similarities in NMR profiles, with key differences that are critical for structure elucidation. Compound **1** was confirmed as rhinocerotinoic acid, consistent with previously reported NMR chemical shifts [28] (Table S1).

Compound **2** showed similarities in NMR chemical shifts compared to **1,** with differences indicating the absence of a double bond at positions 13–14. The notable observation for structural elucidation was the absence of a signal for H-14 at the deshielding area (*δ*_H_ 5.76) compared to **1**, which shifted to a more shielding area at *δ*_H_ 2.24 and 2.37, correlating with *δ*_C_ 40.7, which was also more shielded than **1** (*δ*_C_ 115.4). Additionally, the C-13 signal changed from *δ*_C_ 162.1 (1) to a more shielded signal at *δ*_C_ 31.1 (**2**) (Table S2). Therefore, **2** was identified as 7-oxo-8-labden-15-oic acid, which was previously isolated from *C. ladanifer* [29,30].

Compound **3**, an amorphous light-yellow solid, has the chemical formula C_20_H_32_O_3_ based on HRESI-MS: *m*/*z* at 343.2248 [M + Na]^+^ calculated for 343.2244. The NMR exhibited ^13^C and ^1^H-NMR spectra that resembled those of compound **1**, with notable variations at positions 7, 9, and 17. At position 7 (*δ*_C_ 74.2, *δ*_H_ 4.39), the signal indicated a CH group bonded to the -OH group instead of the C=O group observed in **1** (*δ*_C_ 200.4). At C-9 (*δ*_C_ 50.4), a stronger shielding effect than in **1** (*δ*_C_ 166.5) suggested the absence of a double bond at C-8 and C-9. Additionally, C-17 (*δ*_C_ 109.9) displayed stronger deshielding than in **1** (*δ*_C_ 11.8), indicating that C-17 is a CH_2_ connected to the cyclohexane at C-8 via a double bond. These correlations were confirmed by HSQC and HMBC (Figures S12 and S13). Consequently, the core structure of **3** was identified as 7-hydroxy-labd-8,13-dien-15-oic acid, which, to the best of our knowledge, is reported here for the first time. The NMR chemical shift is presented in Table S3.

**Figure 6:**
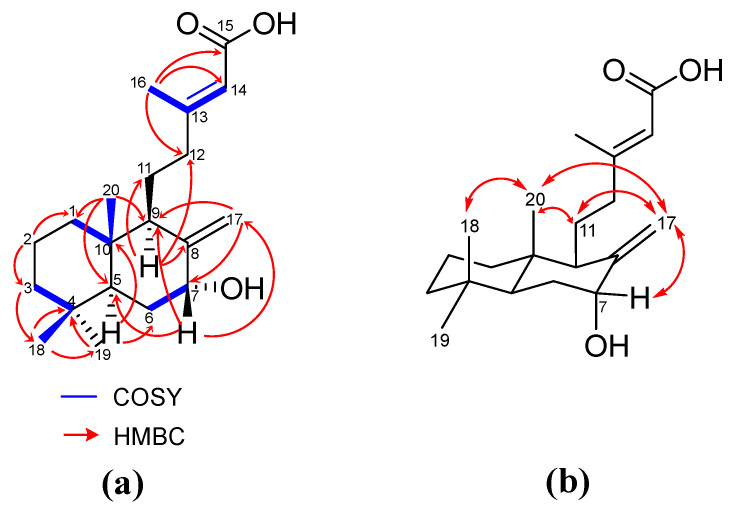
(**a**) Main COSY and HMBC correlations of 3; (**b**) main NOESY correlations of **3**.

**Figure 7:**
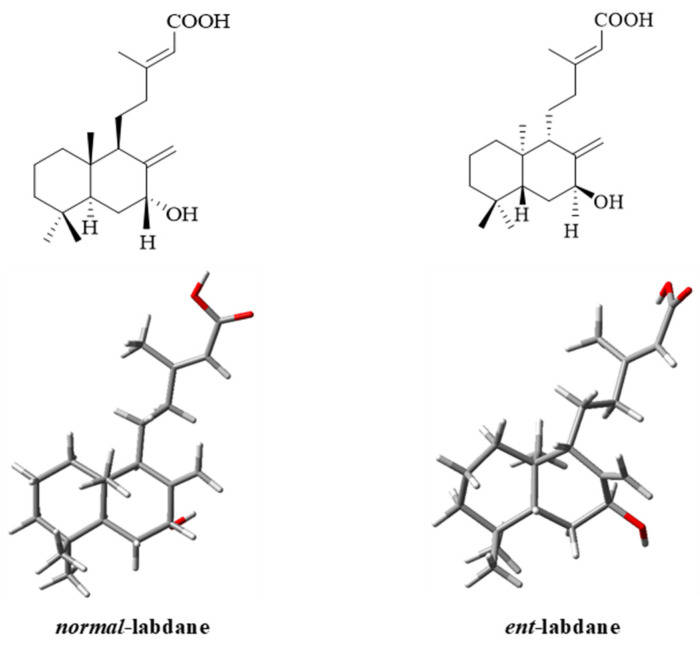
Optimized structures of **3** using B3LYP/6-311++G(d,p) theoretical level.

**Figure 8:**
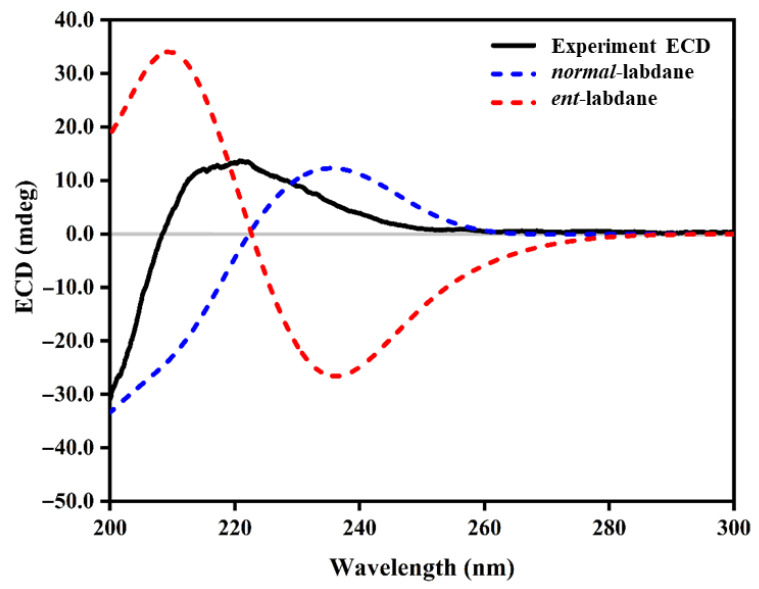
Experimental and calculated ECD spectra of **3**.

The main HMBC correlations of **3** are shown in Figure 6a, and further analysis of NOESY was performed to prove its configuration (Figure 6b). The NOESY spectrum (Figure S15) revealed key correlations in the 4–6 ppm range, while the upfield region showed overlapping signals, which complicated the identification due to the limited amount of isolated material. The observed NOESY correlations, including H-7 (*δ*_H_ 4.39), H-11 (*δ*_H_ 1.49, *δ*_H_ 1.75), H-17 (*δ*_H_ 4.63, *δ*_H_ 5.08), H-18 (*δ*_H_ 0.80), and H-20 (*δ*_H_ 0.67), suggested two possible configurations: *normal*-labdane or *ent*-labdane (Figure 7). Subsequently, the absolute configuration of **3** was confirmed through electronic circular dichroism (ECD) analysis. Calculated ECD spectra for both the *normal*-labdane and *ent*-labdane configurations were generated and compared to the experimental spectrum. The experimental spectrum aligned with the calculated spectrum for the *normal*-labdane configuration, confirming the absolute configuration of **3** (Figure 8).

Compound **4** revealed key NMR signals for identification in comparison with **1**, particularly at positions 7, 8, 9, and 17. At position 7, the carbon signal at *δ*_C_ 44.9 in **4** (vs. *δ*_C_ 200.4 for **1**) indicated the absence of a carbonyl group. Additionally, C-8 and C-9 appeared more shielded in **4** (*δ*_C_ 74.5 and 61.5, respectively) than in **1** (*δ*_C_ 130.9 and 166.5, respectively), confirming that C-8 was bonded to C-9 via a single bond. The signal at *δ*_C_ 74.5 for C-8 further suggested the presence of a hydroxy group and a methyl group (C-17, *δ*_C_ 24.2) attached to this position. These correlations were confirmed through HSQC and HMBC analysis (Figures S22 and S23). Therefore, **4** was identified as 8-hydroxy-labd-13-en-15-oic acid, with NMR chemical shifts matching those reported in the literature [31,32]. However, the referenced studies referred to this compound as labd-13-en-8-ol-15-oic acid, which may represent a deviation from the correct IUPAC nomenclature [31,32]. Notably, Baratta et al. [31] and Monteiro et al. [32] previously isolated and characterized this compound, although they reported different stereochemical configurations. The distinguishing factor was the optical rotation, which exhibited negative and positive values in their respective studies. Compound **4** displayed a negative optical rotation ([$\alpha_{D}^{25}$] −32, *c* 1.25, MeOH), aligning with the configuration proposed by Baratta et al. [31]. However, the absolute configuration could not be confirmed due to the lack of detailed stereochemical data at position 9.

### 2.5. Evaluation of Steroid 5-Alpha Reductase Inhibitory Activity of Identified Compounds

The results of the S5AR inhibitory activity of these candidate compounds are shown in Table 1. Compound **3** displayed the highest S5AR inhibitory activity among the isolated compounds, with an IC_50_ value of 21.93 ± 0.67 µM, followed by **1** and **4**, which had IC_50_ values of 54.16 ± 1.73 µM and 58.17 ± 0.62 µM, respectively. Compound **2** showed moderate inhibition (41.41 ± 1.69% at the screening concentration of 300 µM) and therefore did not undergo further IC_50_ evaluation. Curcumin was used as a positive control and exhibited 92.73 ± 0.14% inhibition at a final concentration of 50 µg/mL. Interestingly, **5** and **6**, which are the major isomeric diterpene compounds in teak leaves, were investigated in the previous study that exhibited the most potent S5AR inhibitory activity, with IC_50_ values around 14 µM [7], which are comparable to that of a naturally occurring reference compound, curcumin (IC_50_ of 13.4 ± 0.4 µM) [26]. Although all teak derived S5AR inhibitors are less potent than finasteride, a clinically used S5AR inhibitor with an IC_50_ of approximately 0.76 µM [26], the effectiveness of a hair growth formulation containing teak leaf extract has been demonstrated in a clinical study [6]. This finding suggests that **5** and **6** could serve as pharmacologically relevant markers for the quality control of teak leaf extracts used in formulations targeting androgen-related conditions such as androgenic alopecia [6,33].

Although the remaining candidate features (F1, F7, F8, and F10) were not successfully isolated and evaluated for bioactivity, they belong to the same class of diterpene compounds. Given the structural similarities, it is plausible that these compounds may also exhibit moderate to high S5AR inhibitory activity. The observed variation in inhibitory performance can be attributed to structural differences, particularly the presence of double bonds at key positions. For example, **1** demonstrated significantly higher inhibitory activity than **2**, despite the two compounds differing only by a single structural feature at the linkage between C13–C14. Compound **1** contains a double bond at this position, whereas **2** has a single bond, suggesting that unsaturation at C13–14 plays a key role in enhancing S5AR inhibition. This observation aligns with previous studies, which have shown that the presence of unsaturated linkages is critical for bioactivity [26,34]. Compared to finasteride, synthetic inhibitors that bind tightly and specifically to the active site of S5AR, the diterpenoids are likely to exert inhibition through a different, potentially less compact binding mode. Finasteride forms a stable complex with NADPH-bound S5AR via its 4-aza structure, mimicking the transition state of testosterone reduction [35]. In contrast, S5AR analysis of the diterpenoids revealed that structural features such as unsaturation at C13–C14 and the presence of a carboxylic acid group at C15 may enhance enzyme interaction.

While these structure–activity relationships suggest potential interaction modes with S5AR, it is important to note that direct binding evidence was not obtained in this study. The enzymatic assay indicates functional inhibition through reduced DHT production; however, it does not confirm physical interaction between the compounds and the enzyme. Further biophysical validation, such as an in silico study or surface plasmon resonance, is required to provide a more comprehensive understanding of the binding mechanisms of these diterpenoids.

## 3. Materials and Methods

### 3.1. General Experiment Procedures

Mass spectrometric (MS) analysis was conducted using an Agilent 1260 Infinity Series HPLC system coupled with an Agilent 6540 UHD Accurate-Mass Q-TOF LC/MS (Santa Clara, CA, USA), operating in dual electrospray ionization (ESI) mode. MS/MS fragmentations were performed at collision energies of 10, 20, and 40 eV. The 1D NMR and 2D NMR spectra were recorded in CDCl_3_ by a Bruker AC400 spectrometer (Bruker BioSpin, Billerica, MA, USA). Chemical shifts (*δ*) are reported in parts per million (ppm) relative to the residual solvent signals (*δ*_H_ 7.26 and *δ*_C_ 77.2). Coupling constants (*J*) are indicated in Hz. The optical rotation values were evaluated using a POLAX-2L polarimeter (Atago, Kyoto, Japan) in MeOH. The UV and ECD spectra were recorded using a J-1100 Circular Dichroism Spectrophotometer (JASCO, Heckmondwike, UK) in acetonitrile. IR spectra were obtained using a Spectrum 65 PerkinElmer with UATR (Waltham, MA, USA), and the principal absorption values were given in cm^−1^. Open-column chromatography was performed using silica 60 (0.063–0.2 mm, 130 g). Flash chromatography (CombiFlash^®^ NEXTGEN 300, Teledyne ISCO, Lincoln, NE, USA) was employed for normal-phase isolation, utilizing a chromabond^®^ (330 g SiOH) stationary phase, while solid-phase extraction (SPE) polypropylene CHROMABOND^®^ C18, 45 µM (Macherey-Nagel GmbH & Co. KG, Düren, Germany), and a reverse-phase C18 column (5 µm, 21.2 × 250 mm) were used for preparative high-performance liquid chromatography (prep HPLC, GILSON PLC 2020, GILSON PLC 2020, Gilson, Inc., Middleton, WI, USA). Organic solvents were of analytical, HPLC, or LC-MS grade, depending on the specific experiment.

### 3.2. Plant Materials

Teak leaves were sourced from 14 provinces in Thailand, categorized into four regions: northern (Chiang Rai, Phayao, Lampang, Phitsanulok), central (Saraburi, Nakhon Nayok, Kanchanaburi, Ratchaburi), northeast (Khon Kaen, Ubon Ratchathani, Buriram), and southern (Surat Thani, Trang, Songkhla). The herbarium specimens were preserved at the Department of Biology, Faculty of Science, Naresuan University, Phitsanulok, Thailand. These specimens were authenticated by Assistant Professor Dr. Pranee Nangngam from the same department. Harvesting occurred every 2–4 months, totaling four times over a year (October 2019, December 2019, April–May 2020, July 2020). The variation in harvesting times was due to the leaf cycle, which typically results in leaf shedding from January to April. The harvested material was classified as young and mature leaves, distinguished by leaf rank: ranks 1–2 were categorized as young leaves, while ranks 3–5 were classified as mature leaves. Eight samples were harvested from the Silvicultural Research Station, while six were obtained from naturally growing teak trees. Further details are provided in Table 2.

### 3.3. Bioactive Compound Extraction

The fresh leaves were dried under 50 °C for 3 days. The dried leaves were ground and sieved (60-mesh), yielding teak leaf powder. The leaf powder was then subjected to extraction using 95% ethanol (*v*/*v*) in a ratio of 1:5 and shaken at 250 rpm for 24 h. After extraction, the solution was filtered through No. 1 Whatman™ filter paper and then the solution was evaporated under low pressure to obtain the crude extract.

### 3.4. Investigation of Chemical Profiles Using LC-MS

The extracts, prepared at 10 mg/mL in methanol, were filtered through a 0.45 µm nylon filter. A QC sample was prepared by mixing 10 µL of each individual sample. The analysis was performed in one batch, and the injection sequence was randomized. The QC sample was monitored for system reproducibility and stability by injection at the beginning, after every 10 samples, and at the end of the analysis. The negative ionization mode was chosen for analysis because it is more suitable for the metabolites of the samples. The MS range was set to 100–1200 *m*/*z* with the optimized parameters, including a gas temperature of 350 °C, a gas flow rate of 10 L/min, and a nebulizer pressure of 30 psig. Mass calibration was performed before beginning the analysis.

The samples were injected at a volume of 10 µL, and separation occurred on a Phenomenex Luna^®^ (Torrance, CA, USA) C18 (2) column (150 mm × 4.6 mm, 5 µm). The gradient started with 50% of 0.1% formic acid in water (solvent A) and 0.1% formic acid in acetonitrile (solvent B). The concentration of solvent B increased linearly from 50% to 100% over 15 min and remained constant until 25 min, followed by a 5 min post-run before starting the next analysis. The flow rate was 1 mL/min. The column temperature was maintained at 35 °C.

### 3.5. Determination of Steroid 5-Alpha Reductase Inhibitory Activity

The S5AR inhibition assay, originally developed by Srivilai et al. [36], quantifies DHT production during the enzymatic conversion of testosterone to DHT, as also presented in previous studies [5,7]. The assay was conducted in a 96-well plate by adding the extract (final assay concentration: 100 µg/mL), testosterone, NADPH, and the S5AR enzyme derived from LNCaP cells (LNCaP clone FGC; American Type Culture Collection (ATCC^®^) CRL-1740™, Manassas, VA, USA). The reactions were incubated at 37 °C for 60 min, then stopped and derivatized DHT by a 60 °C incubation with hydroxylamine dissolved in EtOH. The completed reactions were further centrifuged, the supernatant was collected, and then the DHT content was analyzed using LC-MS. Curcumin was used as positive control. The S5AR inhibitory activity percentage was calculated using the AUC of DHT, as shown in the following equation:

$$5\;\mathrm{alpha}\;\mathrm{reductase}\;\mathrm{inhibitory}\;\mathrm{activity}\left(\%\right)=\left[1-\frac{AUC\;of\;sample-AUC\;of\;C_{0}}{AUC\;of\;C_{60}-AUC\;of\;C_{0}}\right]\times \;100$$

where

AUC_sample = peak area of DHT from the test sample.

AUC_C_0_ = peak area of DHT from the negative control terminated before incubation (representing 0% enzymatic activity).

AUC_C_60_ = peak area of DHT from the control incubated for 60 min (representing 100% enzymatic activity).

Control conditions:

C_0_ (negative control) was terminated prior to incubation to represent the baseline with no enzymatic activity.

C_60_ (positive control) was terminated after 60 min of incubation to represent the maximum enzymatic conversion of testosterone to DHT.

The reactions of each sample were performed in triplicate.

### 3.6. Multivariate Analysis

The raw data of the chemical profiles from LC-MS chromatograms underwent conversion into the mzXML file format. This conversion process was carried out using the MSConvert software version 3.0 (ProteoWizard). Following this, the converted files were transferred to MZmine version 2.53. The prepared data began with (1) mass detection in centroid mode, employing a noise level set at 2 × 10^2^. (2) Chromatogram building utilized the APAP module with a minimum height of 1 × 10^3^, *m*/*z* tolerance 0.025 or 5 ppm. (3) Deconvolution of chromatograms involved the application of wavelets (ADAP) with a signal-to-noise (S/N) threshold of 30, a minimum feature height of 2 × 10^4^, a coefficient/area threshold of 110, a peak duration range of 0.01–1.00, and an RT wavelet range of 0.00–0.05. (4) Deisotoping was conducted with an *m*/*z* tolerance of 0.025 or 5 ppm, an RT tolerance of 0.05 min, and a maximum charge of 2. (5) Chromatograms were aligned using the join aligner with an *m*/*z* tolerance of 0.025 or 5 ppm and an RT tolerance of 0.05 min. (6) Any missing metabolites were addressed through gap-filling with an *m*/*z* tolerance of 0.025 or 5 ppm. (7) The disposition of the duplicate feature was set to *m*/*z* tolerance 0.025 or 5 ppm and RT tolerance 0.05 min. Finally, the table containing *m*/*z* and RT with the intensity of the detected metabolites from all samples was obtained and transferred to the process of multivariate analysis.

The multivariate analysis was performed using SIMCA-P by Umetrics (version 13.0.3.0, Umeå, Sweden). PCA was generated from the information on the chemical composition of teak leaves as an X variable. OPLS analysis was performed by incorporating an X variable with S5AR inhibitory activity as the Y variable. Both PCA and OPLS utilized a Pareto-scaling model. The validity and reliability were evaluated based on R^2^ (goodness of fit) and Q^2^ (predictive ability) values. Both values were close to 1, supporting the model’s validity and robustness. An S-plot was generated from the OPLS score plot, and candidate S5AR inhibitors were selected based on the p[1] and p(corr)[1] values of the features.

### 3.7. Targeted Purification of Candidate S5AR Inhibitors

The teak leaf extract (64.75 g) was homogenized with silica 60 (0.063–0.2 mm, 130 g) and then eluted by using a mixture of 70% hexane and ethyl acetate (EtOAC) until the solution was clear, providing the enriched fraction of candidate compounds, designated as fraction A (20.03 g, 30.93%).

Fraction A (10 g homogenized with 10 g of silica 60) was further subjected to isolation via flash chromatography employing a stationary phase of 330 g of silica. This separation process involved a gradient elution starting with 90% hexane mixed with EtOAC, held constant for 2 min, then increased to 20% EtOAC within 4 min, gradually increasing polarity up to 100% EtOAC within 8 min, and further held constant for 6 min. The flow rate was 300 mL/min, and the total run time was 20 min. The resulting sub-fractions were collected every 2 min, providing 10 sub-fractions. The chemical profile of these sub-fractions was investigated by thin-layer chromatography and HPLC. The target zone was identified in sub-fractions A7 and A8. The separation was repeated 2 times to complete all amounts of fraction A.

Fraction A7 (1.18 g) was subsequently isolated by prep HPLC utilizing a reverse-phase C18 column. The mobile phase employed an isocratic 45% solution of 0.1% formic acid in acetonitrile. The detector was set at 220 nm. The auto collection was set at a minimal 30 mV. Cpd. **1** (39.30 mg) was successfully isolated from this fraction.

Fraction A8 (1.24 g) was subsequently isolated by being loaded onto the SPE (C18) column. The column volume/adsorbent weight was 6 mL/1000 mg. The mobile phase consisted of a gradient of 0.1% formic acid in water (solvent A) and 0.1% formic acid in methanol (solvent C). The separation was performed by pouring 20 mL of the mobile phase mixture, starting with 60%, 80% of solvent C, followed by 40 mL of 100% of solvent C, yielding fractions A8/G1 to G3.

Fraction A8/G2 (354.40 mg) was further purified using the prep HPLC. The mobile phase employed was an isocratic 66% solution of solvent C. The detector was set at 220 nm. The auto collection was set at a minimal 30 mV. Cpd. **2** (19.1 mg), Cpd. **3** (14.2 mg), and Cpd. **4** (21.7 mg) were successfully isolated from this fraction.

*7-hydroxy-labd-8,13-dien-15-oic acid* (**3**): an amorphous light-yellow solid; [$\alpha_{D}^{25}$] −71.4 (*c* 0.7, MeOH); UV (ACN) λ_max_ (log ε) 219.05 nm. (3.92); FT-IR (CH_2_Cl_2_) ν_max_: 3401, 2927, 1692, 1648 cm^−1^; ^1^H-NMR (CDCl_3_, 400 MHz) 5.68 (1H, s, H-14), 5.08 (1H, s, H-17b), 4.63 (1H, s, H-17a), 4.39 (1H, br s, H-7), 2.17 (3H, d, *J* = 1.3 Hz, H-16), 2.29 (1H, m, H-12b), 2.10 (1H, dd, *J* = 12.1, 1.3, H-9), 2.01 (1H, m, H-12a), 1.88 (1H, dd, *J* = 11.8, 2.5 Hz, H-6b), 1.75 (1H, m, H-11b), 1.72 (1H, m, H-1b), 1.63 (1H, m, H-6a), 1.60 (1H, m, H-5), 1.56 (2H, m, H-2), 1.49 (1H, m, H-11a), 1.43 (1H, m, H-3b), 1.22 (1H, m, H-3a), 1.07 (1H, m, H-1a), 0.88 (3H, s, H-19), 0.80 (3H, s, H-18), 0.67 (3H, s, H-20); ^13^C NMR (100 MHz, CDCl_3_) 170.7 (C, C-15), 163.7 (C, C-13), 149.5 (C, C-8), 114.7 (CH, C-14), 109.9 (CH_2_, C-17), 74.2 (CH, C-7), 50.4 (CH, C-9), 47.8 (CH, C-5), 42.2 (CH_2_, C-3), 40.0 (C, C-10), 39.7 (CH_2_, C-12), 38.9 (CH_2_, C-1), 33.4 (CH_3_, C-19), 33.3 (C, C-4), 31.1 (CH_2_, C-6), 21.7 (CH_3_, C-18), 21.1 (CH_2_, C-11), 19.3 (CH_3_, C-16), 19.5 (CH_2_, C-2), 13.6 (CH_3_, C-20); HRESI-MS: *m*/*z* 343.2248 [M + Na]^+^ (calcd C_20_H_32_NaO_3_^+^, 343.2244).

### 3.8. Computational Details

Geometry optimizations were performed using density functional theory (DFT) at the B3LYP/6-311++G(d,p) level, with dispersion effects incorporated through Grimme’s D3 correction with Becke–Johnson damping (GD3BJ). Time-dependent DFT (TD-DFT) calculations at the same level of theory were applied to compute the ECD spectra. All calculations employed the conductor-like polarizable continuum model (CPCM) with acetonitrile as the solvent. Rotary strengths were evaluated for 120 excited states, and the ECD spectra were simulated using a Gaussian band shape with a 0.30 eV bandwidth. The computations were carried out using the Gaussian 16 software package, and theoretical ECD curves were processed and visualized with SpecDis 1.71 (University of Würzburg, Würzburg, Germany).

## 4. Conclusions

In conclusion, this study applied a metabolomics approach to investigate factors influencing the chemical composition and S5AR inhibitory activity of teak leaf extracts. Geographic origin emerged as the primary determinant of bioactivity, while leaf age and seasonal variations had minimal impact, underscoring the importance of sourcing consistency for quality control. Growing teak in locations with proven high-quality chemical profiles would support year-round harvesting of both young and mature leaves. In addition, narrowing future investigations to high-activity sources may further clarify the roles of leaf age and seasonal harvesting in bioactivity outcomes.

Using OPLS analysis, six diterpenoid inhibitors were identified, including a novel diterpene, 7-hydroxy-labd-8,13-dien-15-oic acid. Structure–activity relationship analysis revealed that unsaturation at C13–14 plays a key role in enhancing S5AR inhibition, offering new insights for future drug development. By integrating PCA for exploratory data analysis and OPLS for predictive modeling, this study successfully linked chemical composition to bioactivity, demonstrating the power of metabolomics in natural product research. These findings not only expand the chemical diversity of bioactive diterpenes but also provide a foundation for optimizing teak leaf extract as a therapeutic agent for S5AR-related conditions.

## Figures and Tables

**Table 2:** The geographical detail of the teak leaves harvested in this study.

No.	Raw Material Source	Herbarium Specimen Number
1	Mueang District, Chiang Rai	06967
2	MaeGar Silvicultural Research Station, Phayao	05966
3	Ngao Silvicultural Research Station, Lampang (Teak Improvement Center)	05959
4	Phitsanulok Silvicultural Research Station, Phitsanulok	05960
5	Sao Hai District, Saraburi	05958
6	Banna district, Nakhon Nayok	05721
7	Central Silvicultural Research Station, Kanchanaburi	05957
8	Mueang District, Ratchaburi	05968
9	Dong Lan Silvicultural Research Station, Khon Kaen	05964
10	Kong Jiam Silvicultural Research Station, Ubon Ratchathani	05963
11	Non Din Daeng District, Burirum	05965
12	BanTaKhun Silvicultural Research Station, Surat Thani	05969
13	ThaugKhaoBamTad Silvicultural Research Station, Trang	05962
14	Hat Yai District, Songkhla	05961

## Data Availability

Data is contained within the article.

## Supplementary Materials

The following supporting information can be downloaded at: https://www.mdpi.com/article/10.3390/molecules30142895/s1. The information on NMR, MS, and IR of isolated compounds **1**–**4** and the MS fragmentation of tentative identification of candidate compounds is provided in the supplementary sheet (PDF). Refs. [28,31,37] are cited in the Supplementary Materials.
