# Supplementary material for: Geographic Influence and Metabolomics-Driven Discovery of 5-Alpha Reductase Inhibitors in Tectona grandis L.f. (Teak) Leaves
Source: Molecules. 2025 Jul 8;30(14):2895. doi: 10.3390/molecules30142895 (PMC12300835; doi:10.3390/molecules30142895)
Supplement: Supplementary file 1 [file molecules-30-02895-s001.zip › molecules-3694820-supplementary.pdf]

Supplementary Information

Geographic Influence and Metabolomics-Driven Discovery of 5-Alpha Reductase Inhibitors in *Tectona grandis* L.f. (teak) Leaves

Nutchaninad Tanuphol [1,2], Corine Girard [2], Prapapan Temkitthawon [1], Nungruthai Suphrom[3] , Nitra Nuengchamnong [4], Tongchai Saesong [1], Kamonlak Insumrong [3], Abdulaziz Wadeng [5], Wiyada Khangkhachit [1], Andy Zedet [2], Ratchadaree Intayot [6], Siriporn Jungsuttiwong [6], Anuchit Plubrukarn [5], Francois Senejoux [2] and Kornkanok Ingkaninan [1,] *

1 Center of Excellence for Natural Health Product Innovation and Center of Excellence for Innovation in Chemistry, Department of Pharmaceutical Chemistry and Pharmacognosy, Faculty of Pharmaceutical Sciences, Naresuan University, Phitsanulok 65000, Thailand; nutchaninad789@gmail.com (N.T.); prapapantem@gmail.com (P.T.); tongchai_saesong@hotmail.com (T.S.); khangkhachit.w@outlook.com (W.K.)

2 Université Marie et Louis Pasteur, EFS, INSERM RIGHT (UMR1098), F-25000 Besançon, France; corine.girard@univ-fcomte.fr (C.G.); andy.zedet@univ-fcomte.fr (A.Z.); francois.senejoux@univ-fcomte.fr (F.S.)

3 Department of Chemistry, Faculty of Sciences, Naresuan University, Phitsanulok 65000, Thailand; suphrom.n1@gmail.com (N.S.); insumrong.k@gmail.com (K.I.)

4 Science Laboratory Center, Faculty of Science, Naresuan University, Phitsanulok 65000, Thailand; nitran@nu.ac.th

5 Department of Pharmacognosy and Pharmaceutical Botany, Faculty of Pharmaceutical Sciences, Prince of Songkla University, Hat-Yai, Songkhla 90112, Thailand; phoogun001@gmail.com (A.W.); anuchit.pl@psu.ac.th (A.P.)

6 Center of Excellence for Innovation in Chemistry, Department of Chemistry, Faculty of Science, Ubon Ratchathani University, Ubon Ratchathani 34190, Thailand; ratchadaree.in.64@ubu.ac.th (R.I.); siriporn.j@ubu.ac.th (S.J.)

* Correspondence: k_ingkaninan@yahoo.com or kornkanoki@nu.ac.th

Contents of supplementary data

Identification of rhinocerotinoic acid(**1**) 4

Table S1 NMR data of **1** (in $CDCl_3$) recorded at 400 (^1H) and 100 (^{13}C) MHz and that of Rhinocerotinoic acid (in $CDCl_3$) from reported data at 400 (^1H), and 100 (^{13}C) MHz 4

Figure S1 ^1H–NMR spectrum of **1** (400 MHz, $CDCl_3$) 5

Figure S2 ^{13}C–NMR spectrum of **1** (DEPT-Q, 100 MHz, $CDCl_3$) 6

Figure S3 IR spectrum of **1** 7

Figure S4 HRESI–MS (negative ion mode) spectrum of **1** 7

Figure S5 HRESI–MS (positive ion mode) spectrum of **1** 7

Identification of 7-oxo-8-labden-15-oic acid (**2**) 8

Table S2 NMR data of **2** (in $CDCl_3$) recorded at 400 (^1H) and 100 (^{13}C) MHz and 1 (in $CDCl_3$) recorded at 400 (^1H), and 100 (^{13}C) MHz 8

Figure S6 ^1H–NMR spectra of **2** (400 MHz, $CDCl_3$) 9

Figure S7 ^{13}C–NMR spectra of **2** (100 MHz, $CDCl_3$) 10

Figure S8 IR spectrum of **2** 11

Figure S9 HRESI–MS (positive ion mode) spectrum of **2** 11

Identification of 7-hydroxy-labd-8,13-dien-15-oic acid (**3**) 12

Table S3 1D and 2D-NMR data for compound **3** (diluted in $CDCl_3$ recorded at 400 MHz for ^1H and 100 MHz for ^{13}C) 12

Figure S10 ^1H–NMR spectra of **3** (400 MHz, $CDCl_3$) 13

Figure S11 ^{13}C–NMR spectra of **3** (DEPT-Q, 100 MHz, $CDCl_3$) 14

Figure S12 HSQC spectra of **3** (400 MHz for ^1H and 100 MHz, for ^{13}C, $CDCl_3$), ... 15

Figure S13 HMBC spectra of **3** (400 MHz for ^1H and 100 MHz, for ^{13}C, $CDCl_3$) 16

Figure S14 COSY spectra of **3** (400 MHz, $CDCl_3$) 17

Figure S15 NOESY spectra of **3** (400 MHz, $CDCl_3$) 18

Figure S16 IR spectrum of **3** 19

Figure S17 HRESI–MS (positive ion mode) spectrum of **3** 19

Figure S18 UV spectrum of **3** (0.05 mg/ml, dissolved in acetonitrile) 20

Figure S19 ECD spectrum of **3** (0.05 mg/ml, dissolved in acetonitrile) 20

Identification of 8-hydroxy-labd-13-en-15-oic acid (**4**) 21

Table S4 NMR data of **4** (in $CDCl_3$) recorded at 400 (^1H) and 100 (^{13}C) MHz and that of Labd-13-en-8-ol-15-oic acid (in $CDCl_3$) from reported data at 250 (^1H), and 62.9 (^{13}C) MHz 21

Figure S20 ^{1}H–NMR spectrum of **4** (400 MHz, $CDCl_3$) 22
Figure S21 ^{13}C–NMR spectrum of **4** (DEPT-Q, 100 MHz, $CDCl_3$) 23
Figure S22 HSQC spectrum of **4** (400 MHz for ^{1}H and 100 MHz, for ^{13}C, $CDCl_3$) 24
Figure S23 HMBC spectrum of **4** (400 MHz for ^{1}H and 100 MHz, for ^{13}C, $CDCl_3$) 25
Figure S24 IR spectrum of **4** 26
Figure S25 HRESI–MS (positive ion mode) spectrum of **4** 26
The MS information of the features identified by the MS-MS technique 27
Table S5 MS-MS fragmentation of 10 features 27
Figure S26 MS-MS fragmentation (positive ion mode) of feature 1 28
Figure S27 MS-MS fragmentation (negative ion mode) of feature 2 28
Figure S28 MS-MS fragmentation (positive ion mode) of feature 3 28
Figure S29 MS-MS fragmentation (positive ion mode) of feature 4 28
Figure S30 MS-MS fragmentation (positive ion mode) of feature 5 29
Figure S31 MS-MS fragmentation (negative ion mode) of feature 7 29
Figure S32 MS-MS fragmentation (positive ion mode; $[M+H-H2O]^+$) of feature 8 29
Figure S33 MS-MS fragmentation (negative ion mode) of feature 9 29
Figure S34 MS-MS fragmentation (negative ion mode) of feature 10 30
Figure S35 MS-MS fragmentation (negative ion mode) of feature 11 30
References 31

Identification of rhinocerotinoic acid(1)

Table S1 NMR data of **1** (in $CDCl_3$) recorded at 400 (^1H) and 100 (^{13}C) MHz and that of Rhinocerotinoic acid (in $CDCl_3$) from reported data at 400 (^1H), and 100 (^{13}C) MHz

Position	Rhinocerotinoic acid [1]		Compound 1	
	δ_C (ppm)	δ_H (ppm)(*J* in Hz)	δ_C (ppm)[a],mult.[b]	δ_H (ppm)[a], mult.(*J* in Hz)
**1**	36.0	1.35 / 1.90	36.3, CH_2	1.35, *m* / 1.91, *m*
**2**	18.6	1.68 / 1.58	18.9, CH_2	1.70, *d* (3.7) / 1.62, *m*
**3**	41.3	1.20 / 1.47	41.6, CH_2	1.22, *m* / 1.48, *m*
**4**	33.1	–	33.5, C	–
**5**	50.3	1.68	50.6, CH	1.72, *d* (3.7)
**6**	35.2	2.49 / 2.35	35.5, CH_2	2.50, *dd* (3.5, 17.5) / 2.35, *m*
**7**	200.1	–	200.4, C	–
**8**	130.6	–	130.9, C	–
**9**	166.2	–	166.5, C	–
**10**	41.0	–	41.4, C	–
**11**	27.7	2.28	28.0, CH_2	2.37, *m**
**12**	39.8	2.35	40.1, CH_2	2.31, *m**
**13**	161.5	–	162.1, C	–
**14**	115.4	5.73	115.4, CH	5.76, *br s*
**15**	171.6	–	171.0, C	–
**16**	19.1	2.20	19.5, CH_3	2.23, *d* (1.1)
**17**	11.4	1.75	11.8, CH_3	1.78, *s*
**18**	32.5	0.86	32.8, CH_3	0.89, *s*
**19**	21.3	0.90	21.6, CH_3	0.92, *s*
**20**	18.2	1.07	18.5, CH_3	1.10, *s*

[a]Assignments were based on ^1H, ^{13}C; [b] Multiplicities were established by DEPT-Q experiment.
*overlapped signals. δ values were measured from the HSQC spectrum.[2]

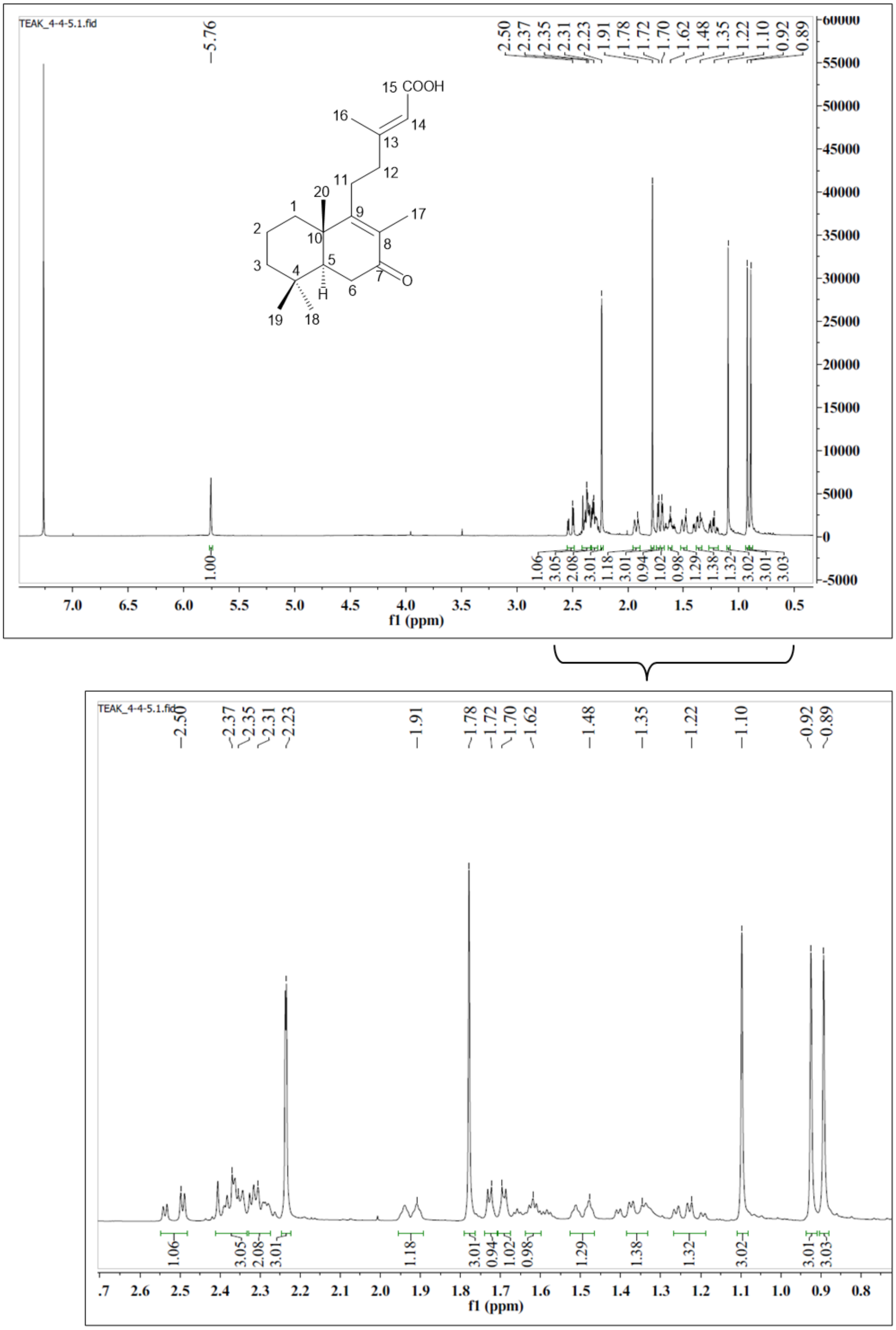

Figure S1 ^{1}H−NMR spectrum of 1 (400 MHz, $CDCl_3$)

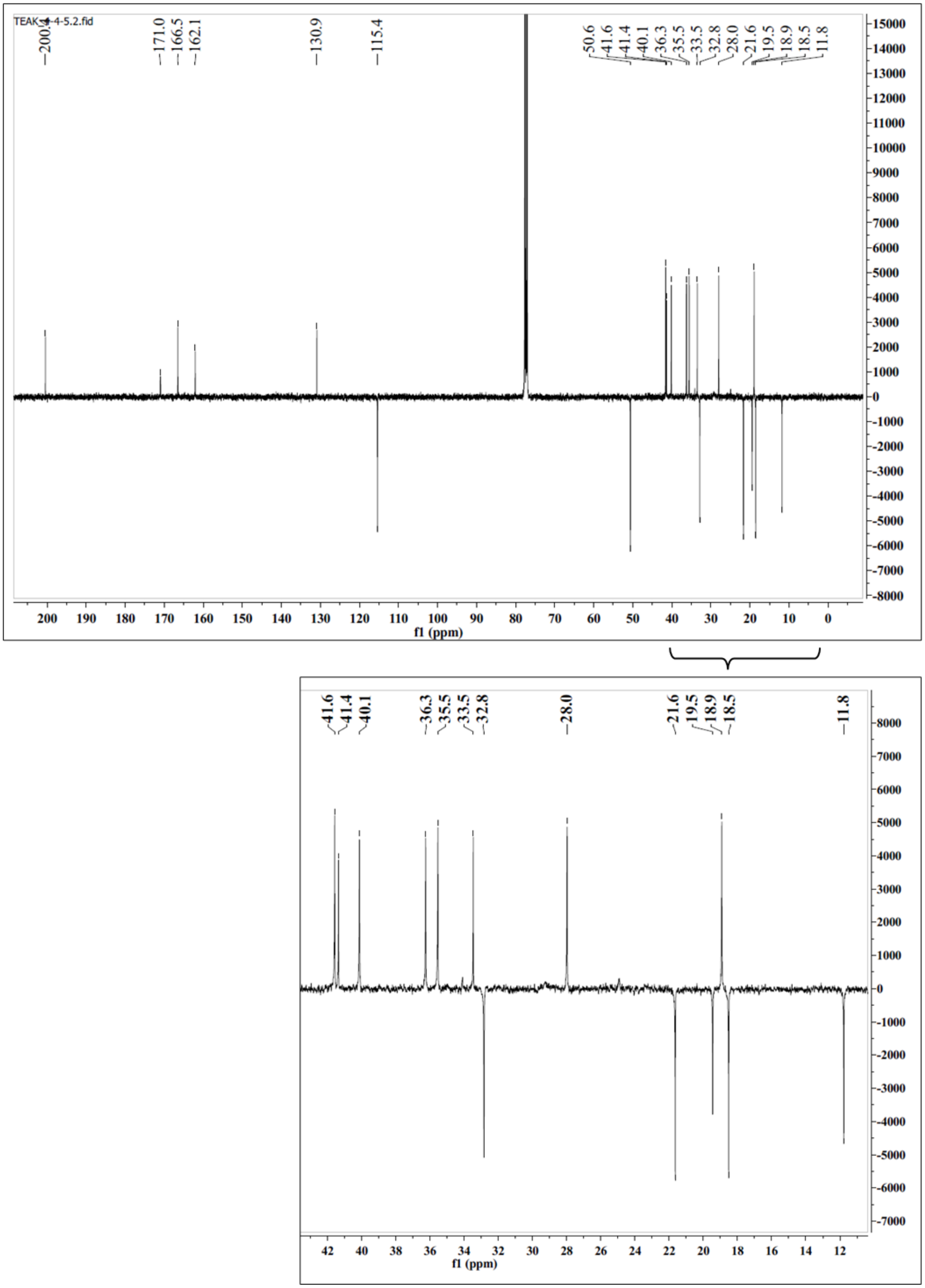

Figure S2 ^{13}C−NMR spectrum of **1** (DEPT-Q, 100 MHz, $CDCl_3$)

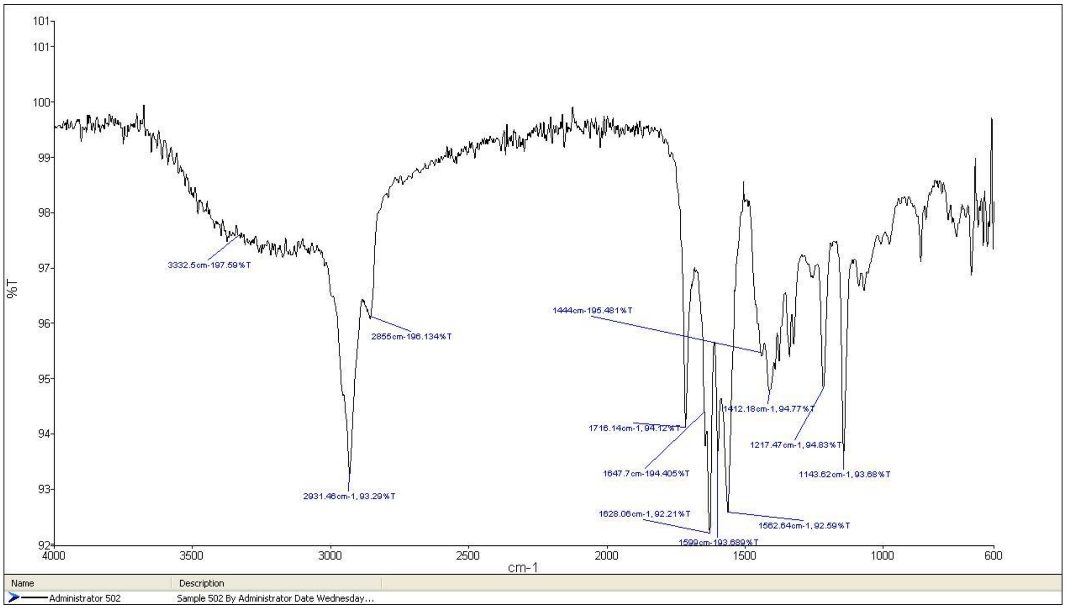

Figure S3 IR spectrum of **1**

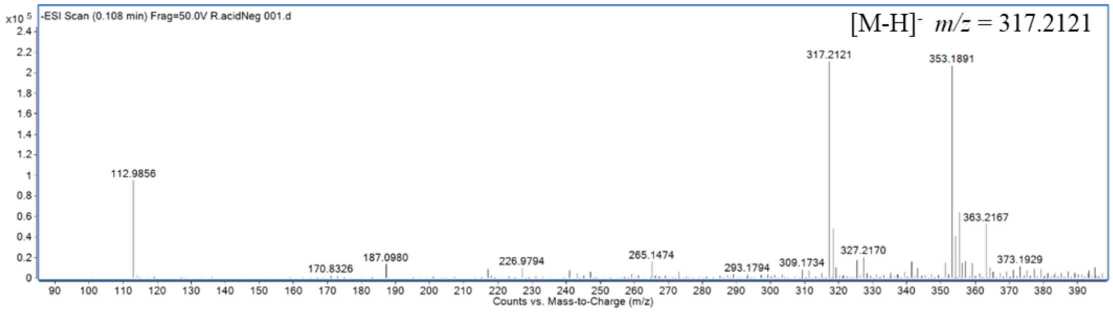

Figure S4 HRESI−MS (negative ion mode) spectrum of **1**

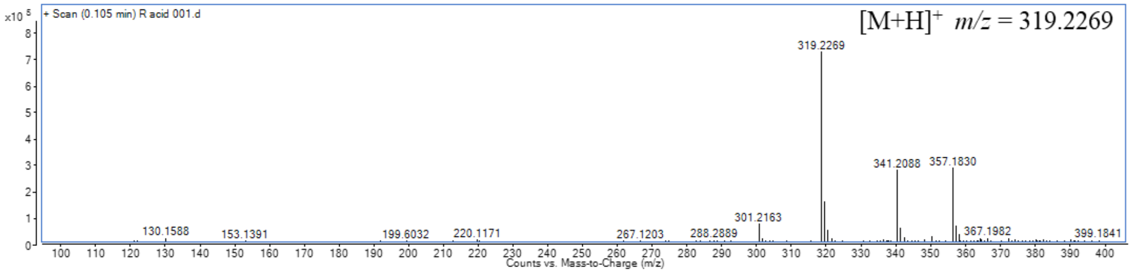

Figure S5 HRESI−MS (positive ion mode) spectrum of **1**

Identification of 7-oxo-8-labden-15-oic acid (2)

Table S2 NMR data of **2** (in $CDCl_3$) recorded at 400 (^1H) and 100 (^{13}C) MHz and **1** (in $CDCl_3$) recorded at 400 (^1H), and 100 (^{13}C) MHz

Position	Compound 1		Compound 2	
	δ_C (ppm)[a],mult.[b]	δ_H (ppm)[a], mult.(*J* in Hz)	δ_C (ppm)[a]	δ_H (ppm)[a], mult.(*J* in Hz)
**1**	36.3, CH_2	1.35, *m* / 1.91, *m*	35.7	1.35,*m*/ 1.90,*m*
**2**	18.9, CH_2	1.70, *d* (3.7) / 1.62, *m*	18.4	1.70,*d* (3.7)/ 1.59,*m*
**3**	41.6, CH_2	1.22, *m* / 1.48, *m*	41.1	1.22,*m*/ 1.49,*m*
**4**	33.5, C	–	33.0	-
**5**	50.6, CH	1.72, *d* (3.7)	50.1	1.67, *d* (3.7)
**6**	35.5, CH_2	2.50, *dd* (3.5, 17.5) / 2.35, *m*	35.0	2.51, *dd* (3.7,17.5)/ 2.35, *m*
**7**	200.4, C	–	200.2	-
**8**	130.9, C	–	129.8	-
**9**	166.5, C	–	168.0	-
**10**	41.4, C	–	40.8	-
**11**	28.0, CH_2	2.37, *m**	26.9	2.39, *m*
**12**	40.1, CH_2	2.31, *m**	35.2	1.46, *m*
**13**	162.1, C	–	31.1	2.04, *m*
**14**	115.4, CH	5.76, *br s*	40.7	2.24, *m* / 2.37, *m*
**15**	171.0, C	–	177.4	-
**16**	19.5, CH_3	2.23, *d* (1.1)	19.3	1.04,*d* (6.7)
**17**	11.8, CH_3	1.78, *s*	11.1	1.74, *s*
**18**	32.8, CH_3	0.89, *s*	32.3	0.88, *s*
**19**	21.6, CH_3	0.92, *s*	21.2	0.91, *s*
**20**	18.5, CH_3	1.10, *s*	18.0	1.07, *s*

[a]Assignments were based on ^1H, ^{13}C; [b] Multiplicities were established by DEPT-Q experiment.

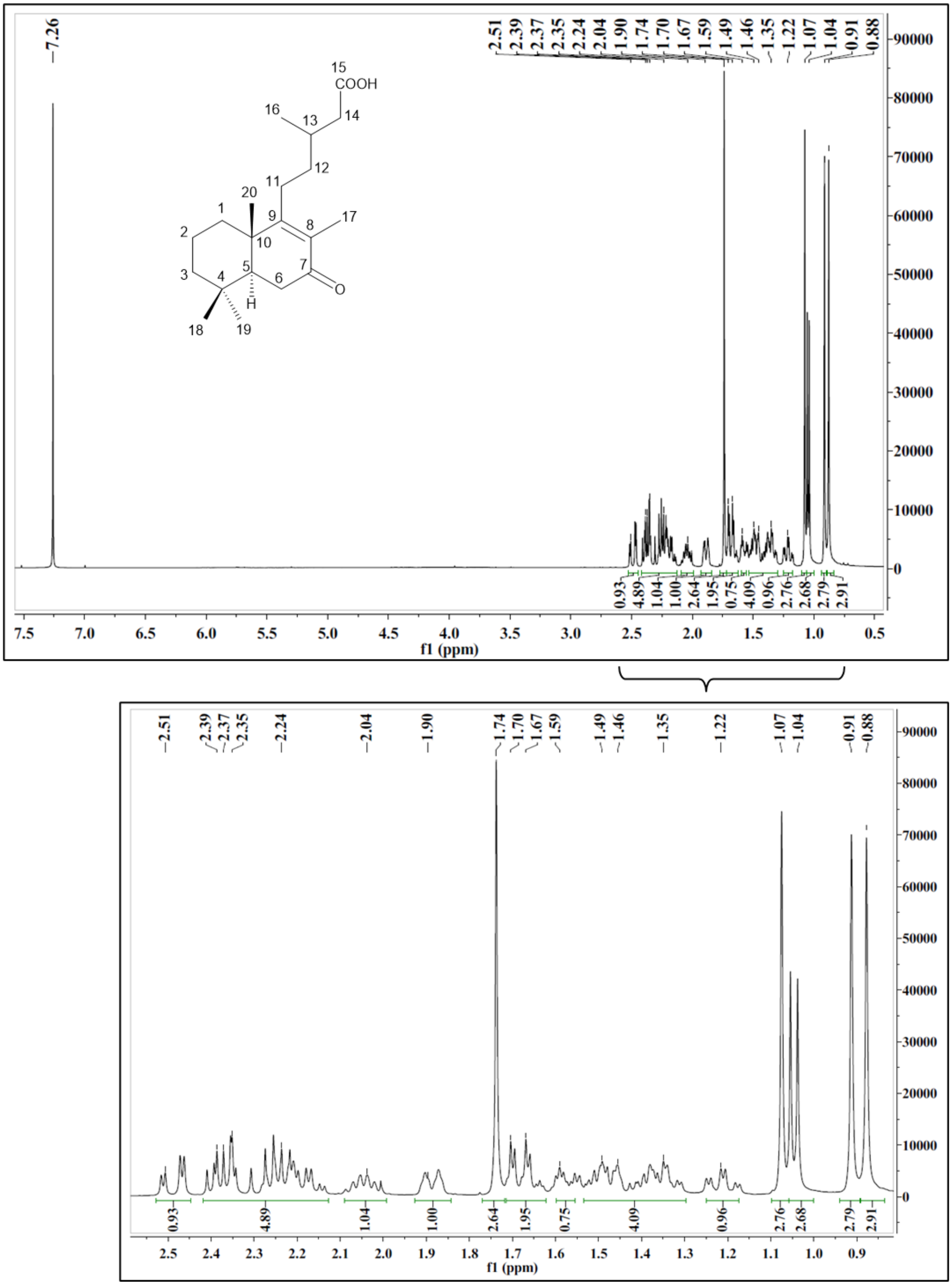

Figure S6 ¹H−NMR spectra of **2** (400 MHz, $CDCl_3$)

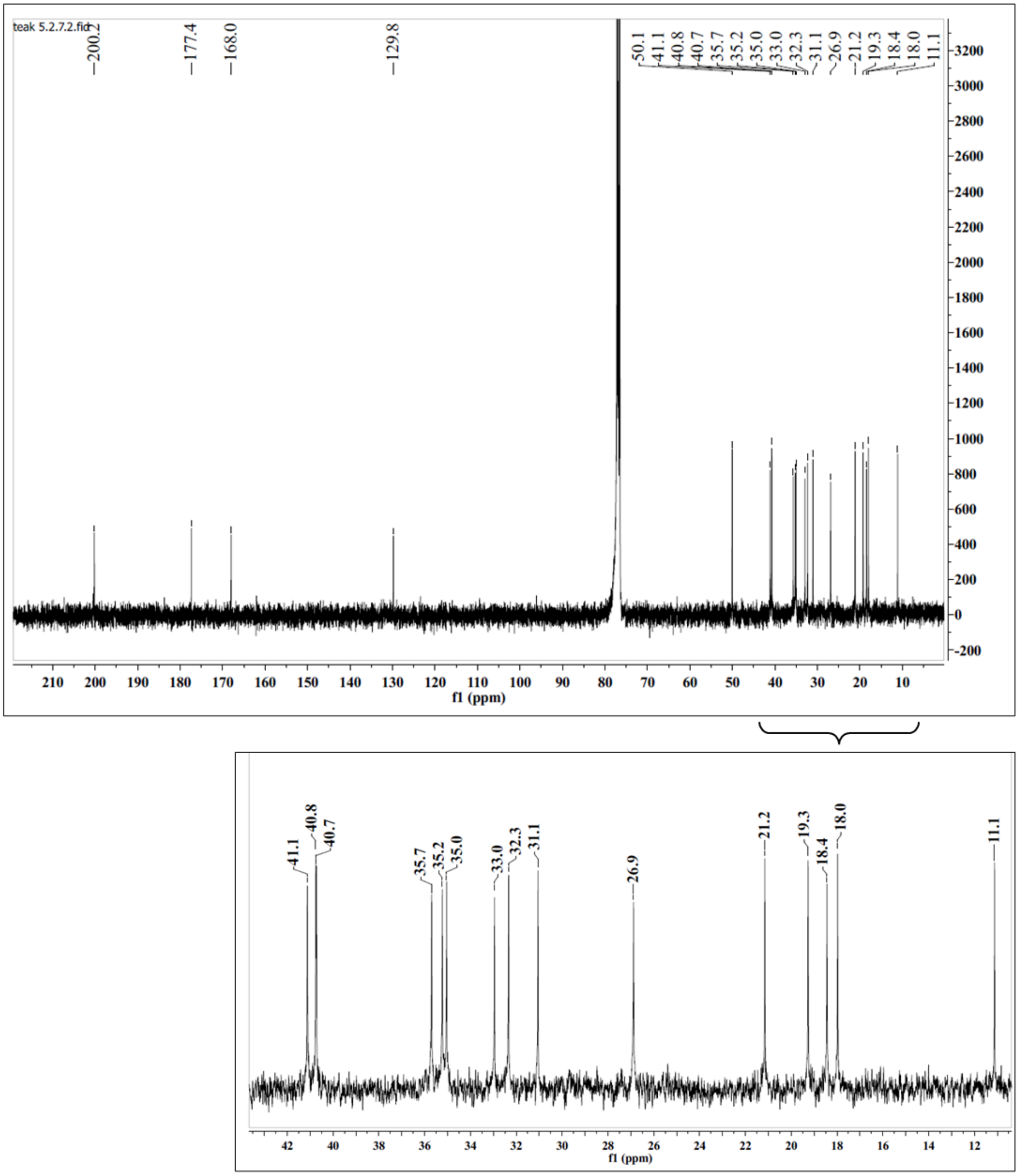

Figure S7 ^{13}C−NMR spectra of **2** (100 MHz, $CDCl_3$)

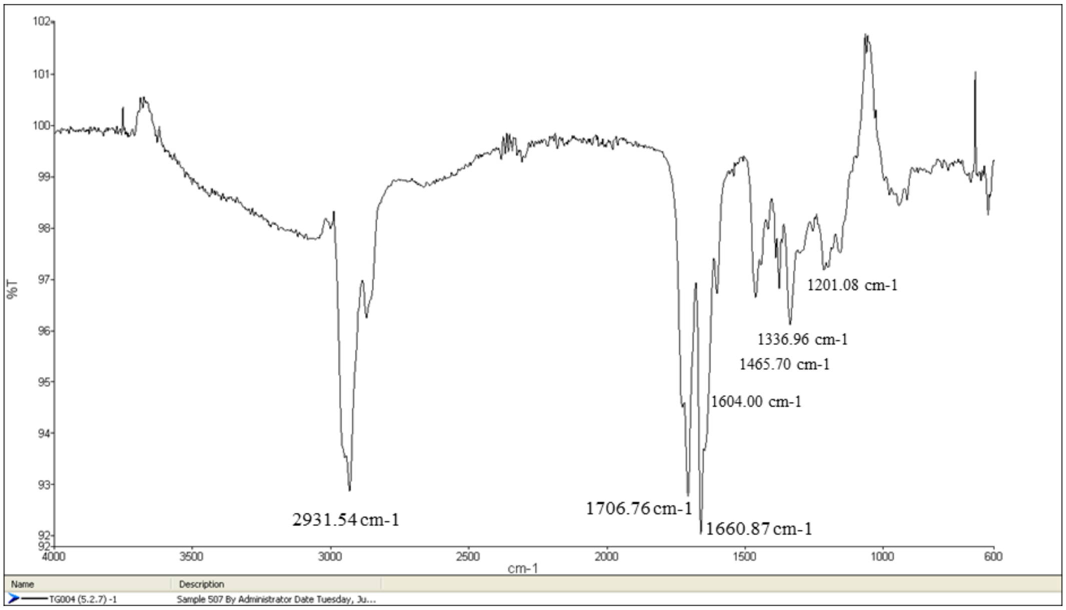

Figure S8 IR spectrum of **2**

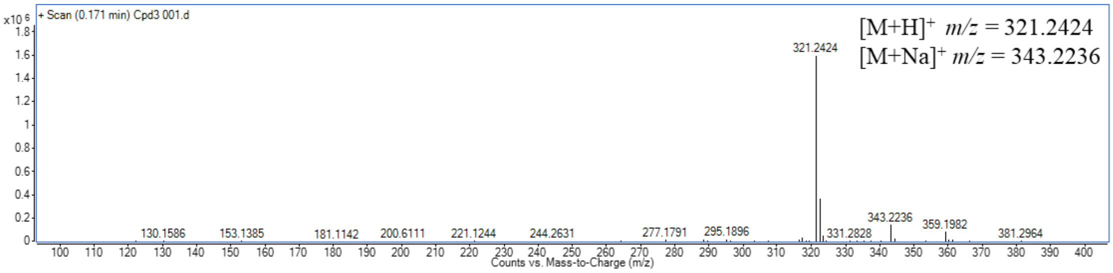

Figure S9 HRESI−MS (positive ion mode) spectrum of **2**

Identification of 7-hydroxy-labd-8,13-dien-15-oic acid (3)

Table S3 1D and 2D-NMR data for compound **3** (diluted in $CDCl_3$ recorded at 400 MHz for ^1H and 100 MHz for ^{13}C)

Position	δ_C (ppm)[a], *mult.*[b]	δ_H (ppm)[a], *mult* (*J* in Hz)	^1H-^1H COSY	HMBC	Key NOESY correlation
1a	38.9, CH_2	1.07,*m*	1b, 2, 20	C-2, 9, 10, 20	
1b		1.72,*m*	1a	C-2, 5, 9, 10, 20	
2	19.5, CH_2	1.56,*m*		C-1, 3,4	
3a	42.2, CH_2	1.22,*m*	2, 3b, 18	C-1, 2, 4, 18, 19	
3b		1.43,*m*	3a	C-4, 5, 18	
4	33.3, C	-		-	
5	47.8, CH	1.60, *m*	6b	C-4, 6, 7, 9, 10, 18, 20	
6a	31.1, CH_2	1.63, *m*	7	C-4, 7, 10, 18	H-20,18
6b		1.88, *dd* (11.8, 2.5)	5,7	C-7, 8	H-19,7
7	74.2, CH	4.39, *br s*	6a, 6b,	C-5, 9, 17	H-20, 6a, 6b
8	149.5, C	-	-	-	
9	50.4, CH	2.10, *dd* (12.1, 1.3)	17a, 17b	C-8, 10, 11, 12, 17, 20	
10	40.0, C	-	-	-	
11a	21.1, CH_2	1.49, *m*	9	C-8, 9, 10, 12, 13	
11b		1.75, *m*	11a, 12b	C-8, 10, 12	
12a	39.7, CH_2	2.01, *m*	11a, 11b	C-11, 13, 14, 16	
12b		2.29, *m*	11b, 12a	C-9,11,14, 16	
13	163.7, C	-	-	-	
14	114.7,CH	5.68, *d (1.1)*	16	C-12, 15, 16	H11a, 11b, 12a, 12b
15	170.7, C	-			
16	19.3, CH_3	2.17, *d* (1.3)	12b, 14	C-12, 13, 14, 15	
17a	109.9, CH_2	4.63, *s*	9, 17b	C-7, 8, 9	H20, H11a, H12a, H12b
17b		5.08, *s*	9, 17a	C-7, 8, 9	H7, H17a
18	21.7, CH_3	0.80, *s*	3b	C-3, 4, 5, 19	H20
19	33.4, CH_3	0.88, *s*		C-3, 5, 4, 18	H6b
20	13.6, CH_3	0.67, *s*	1a, 9	C-1, 5, 9, 10	H 7, H6a

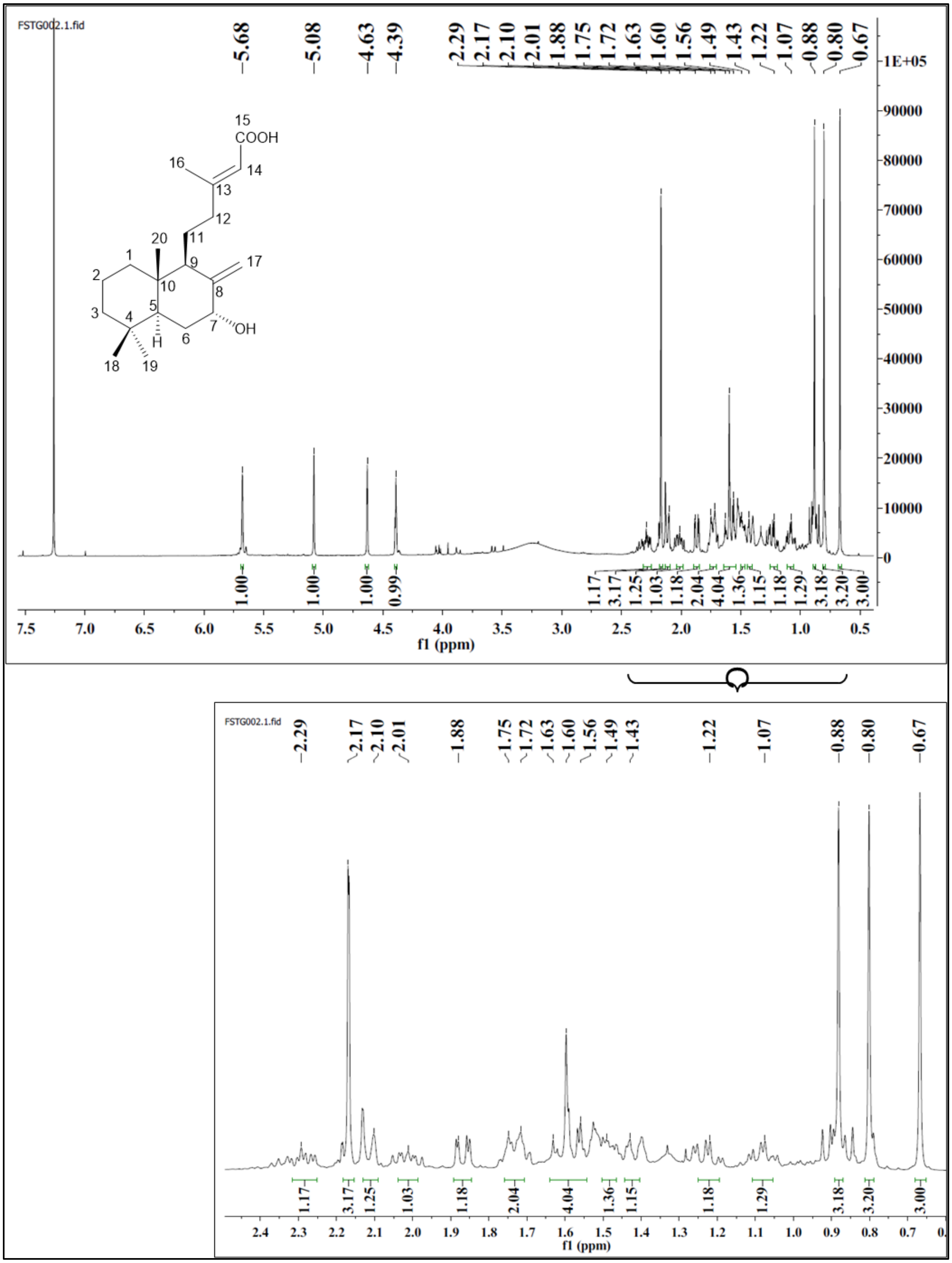

Figure S10 ^{1}H−NMR spectra of **3** (400 MHz, CDCl$_3$)

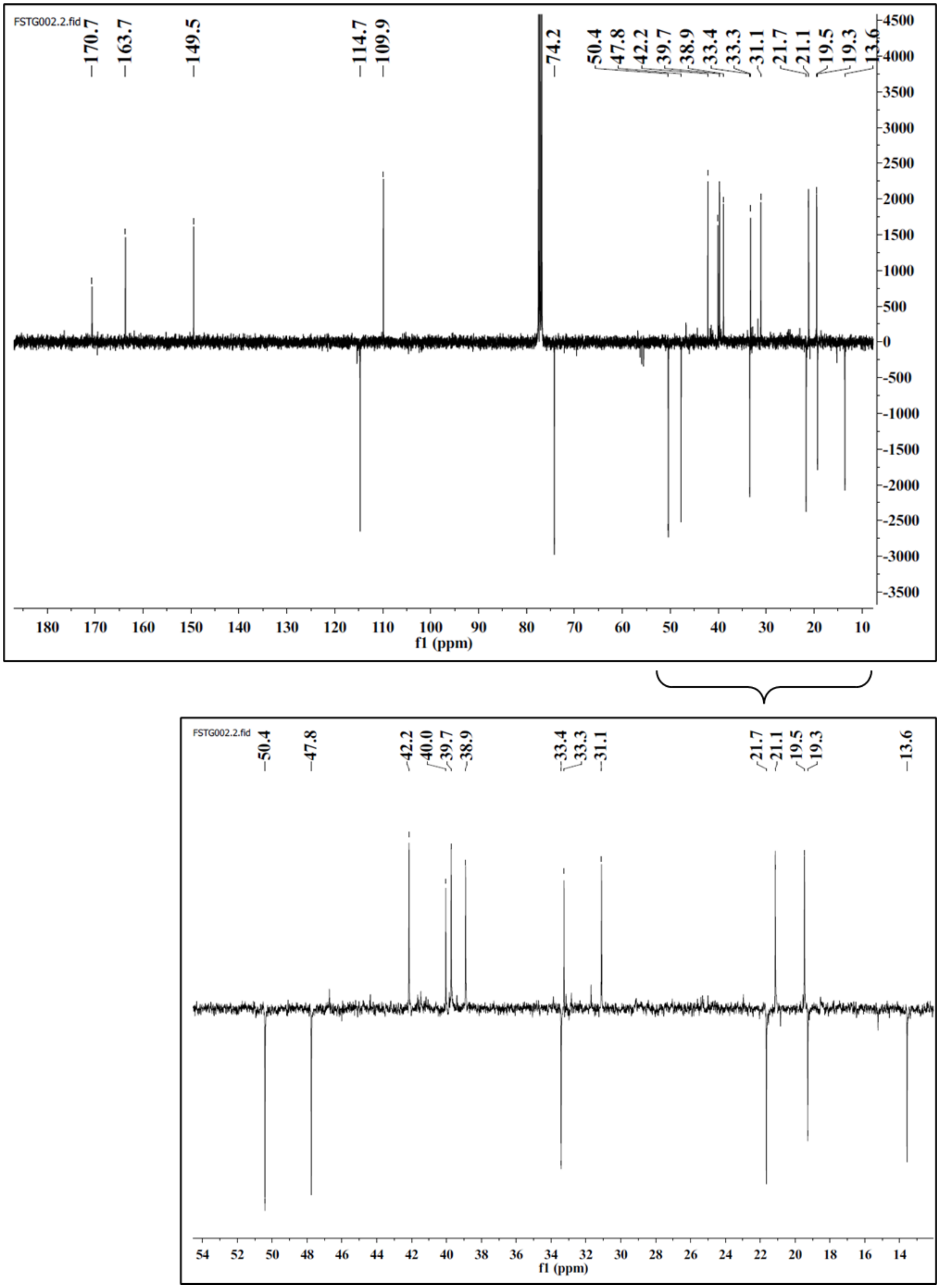

Figure S11 ^{13}C−NMR spectra of **3** (DEPT-Q, 100 MHz, $CDCl_3$)

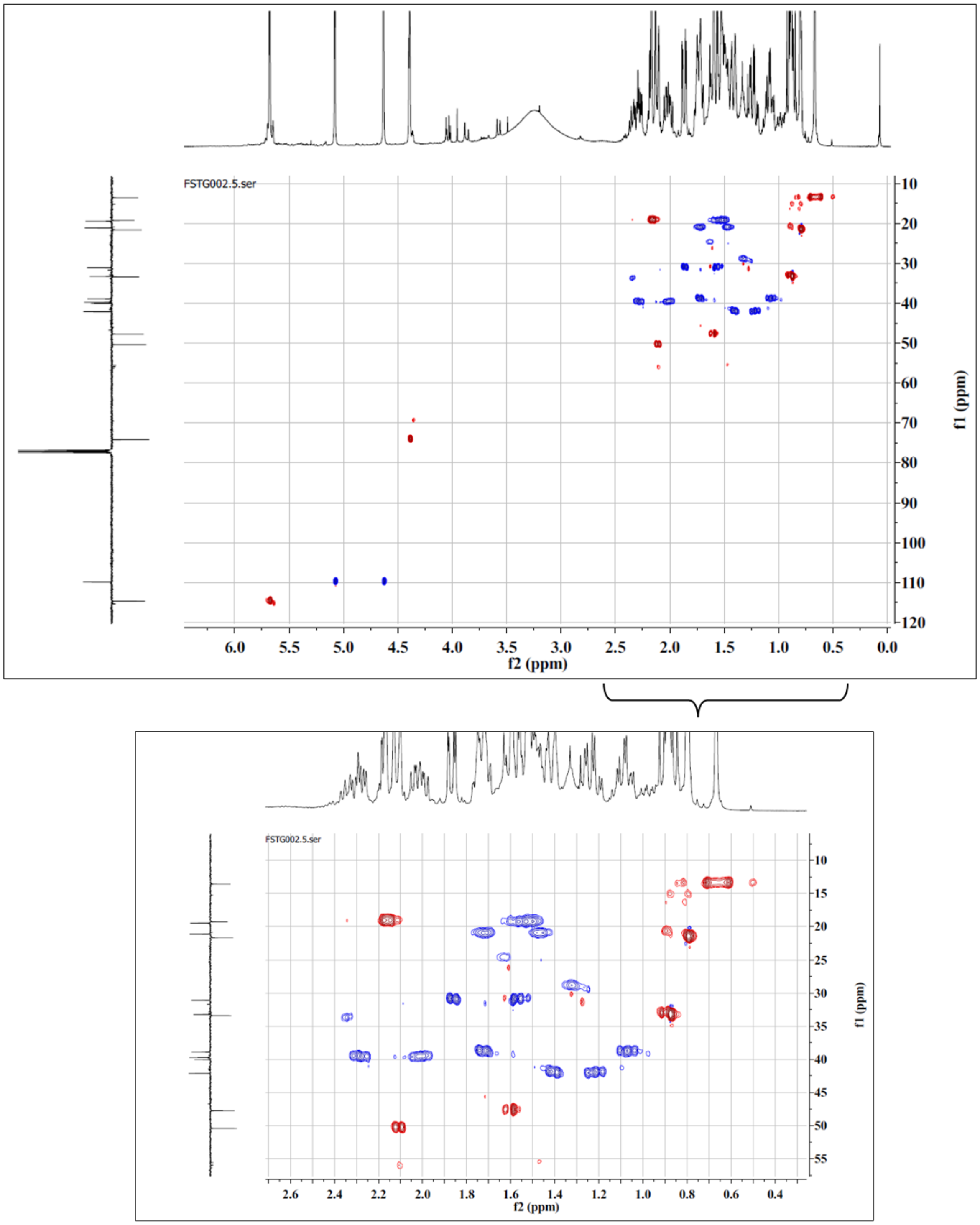

Figure S12 HSQC spectra of **3** (400 MHz for ^{1}H and 100 MHz, for ^{13}C, $CDCl_3$),

(Blue spot; C, CH_2/Red spot; CH, CH_3)

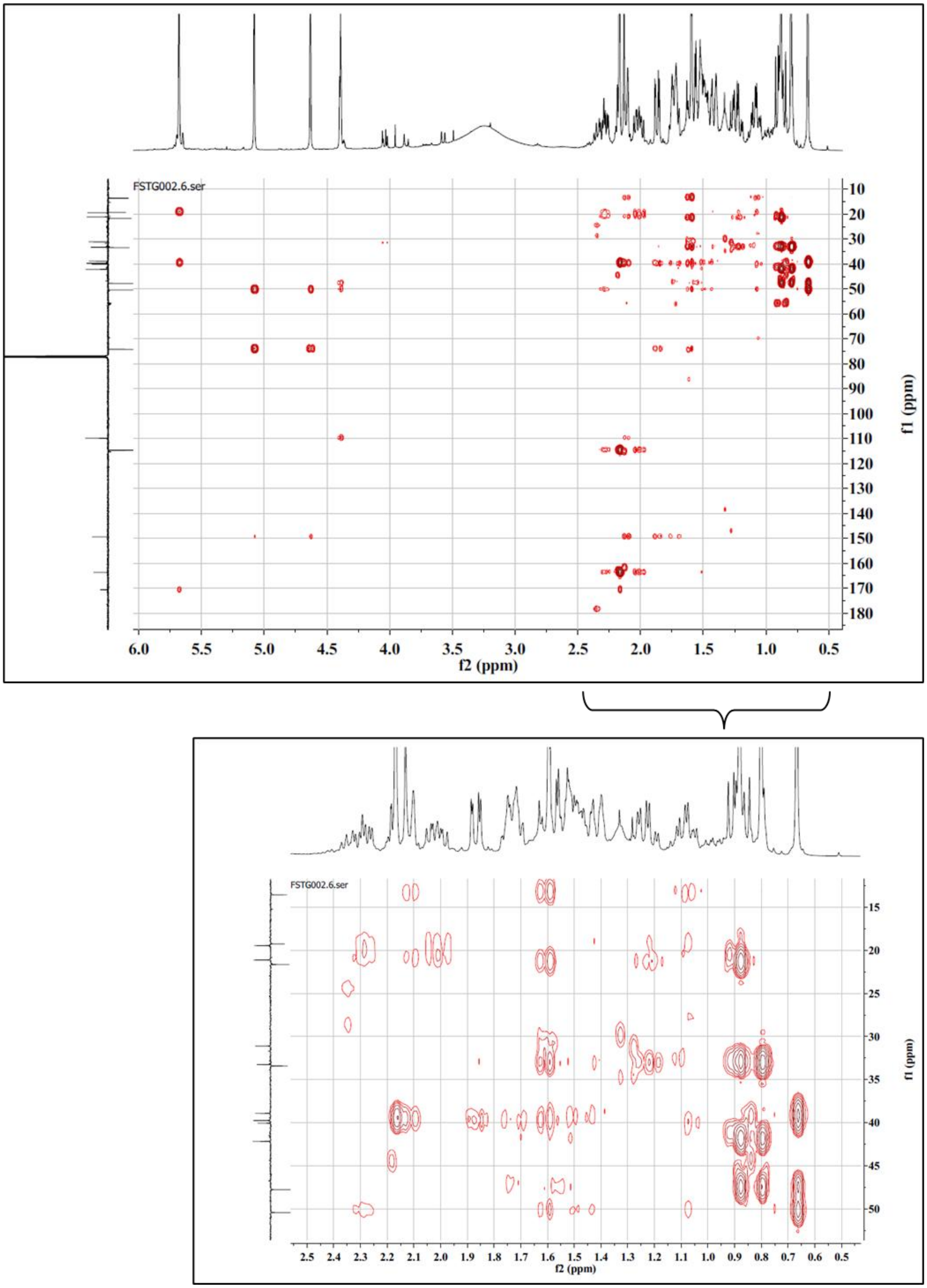

Figure S13 HMBC spectra of **3** (400 MHz for ^{1}H and 100 MHz, for ^{13}C, $CDCl_3$)

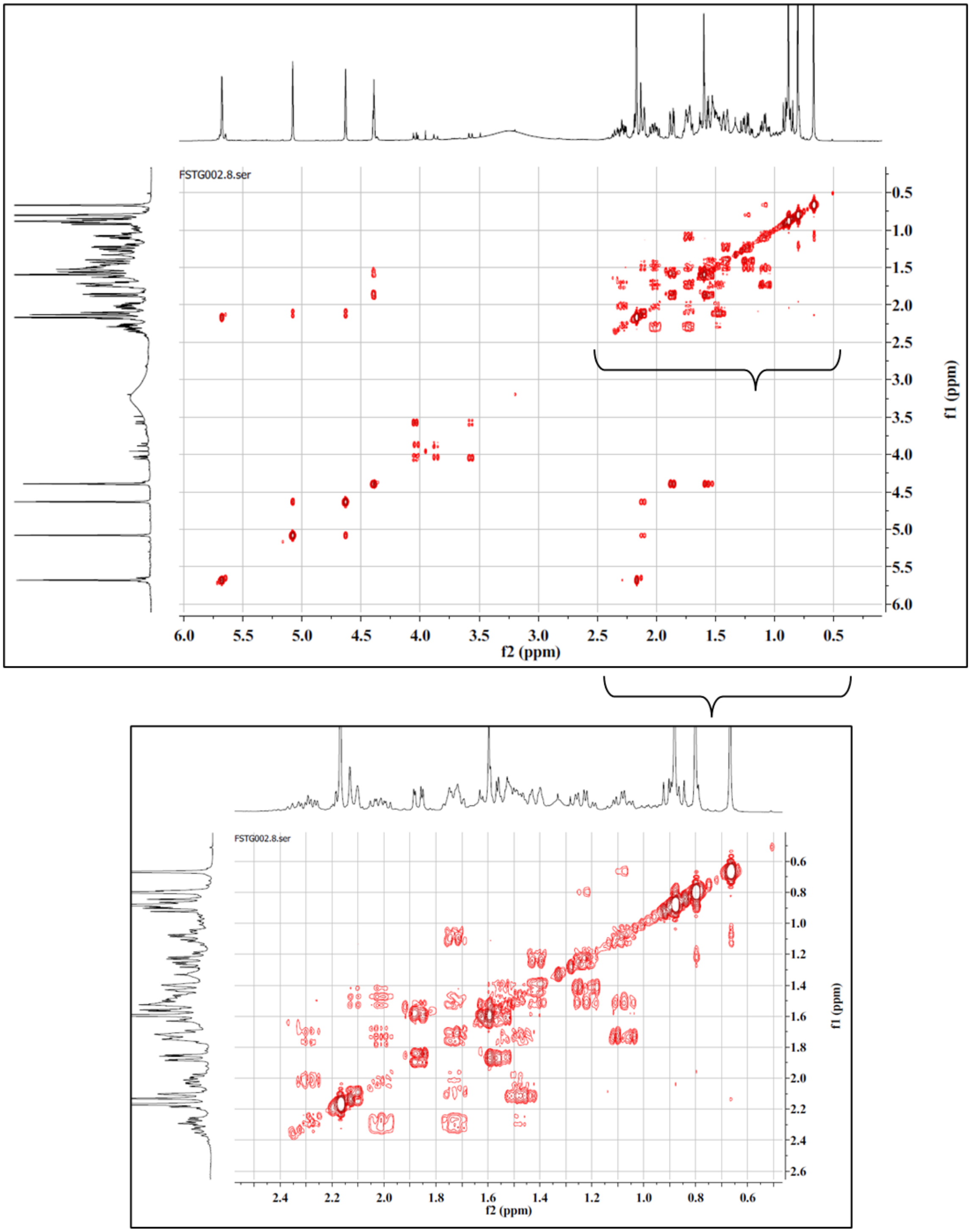

Figure S14 COSY spectra of **3** (400 MHz, $CDCl_3$)

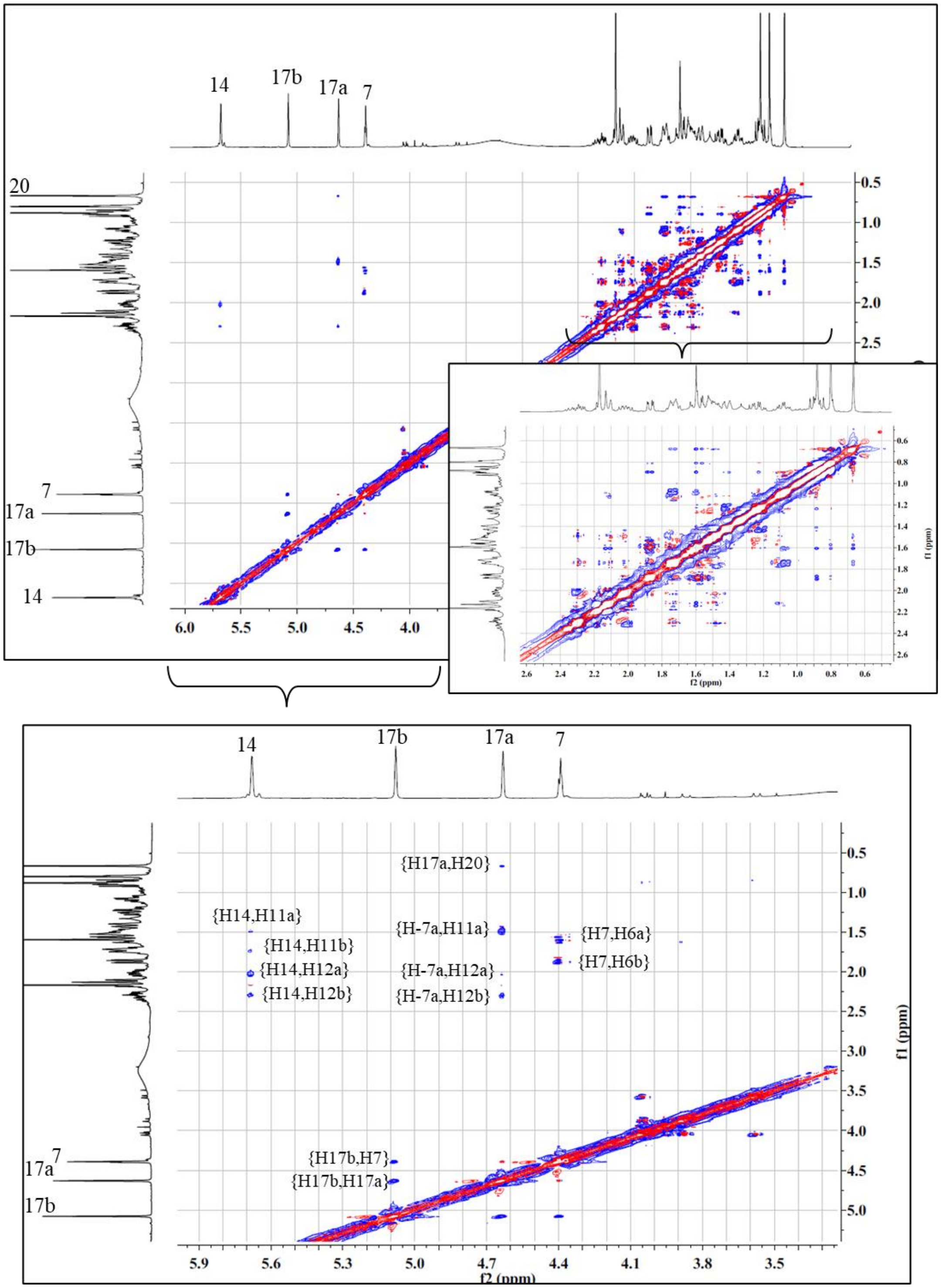

Figure S15 NOESY spectra of **3** (400 MHz, $CDCl_3$)

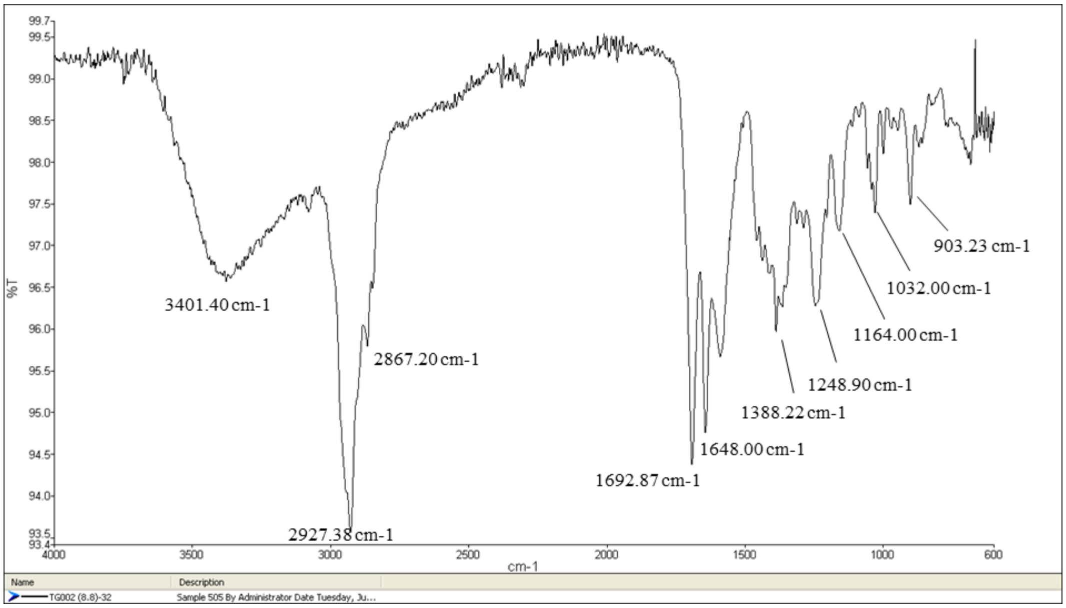

Figure S16 IR spectrum of **3**

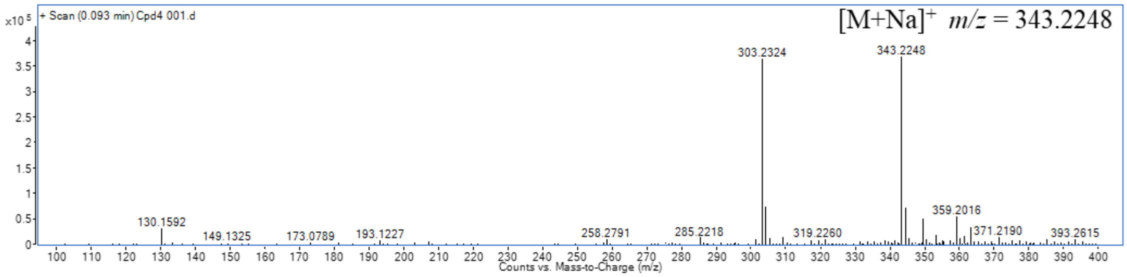

Figure S17 HRESI−MS (positive ion mode) spectrum of **3**

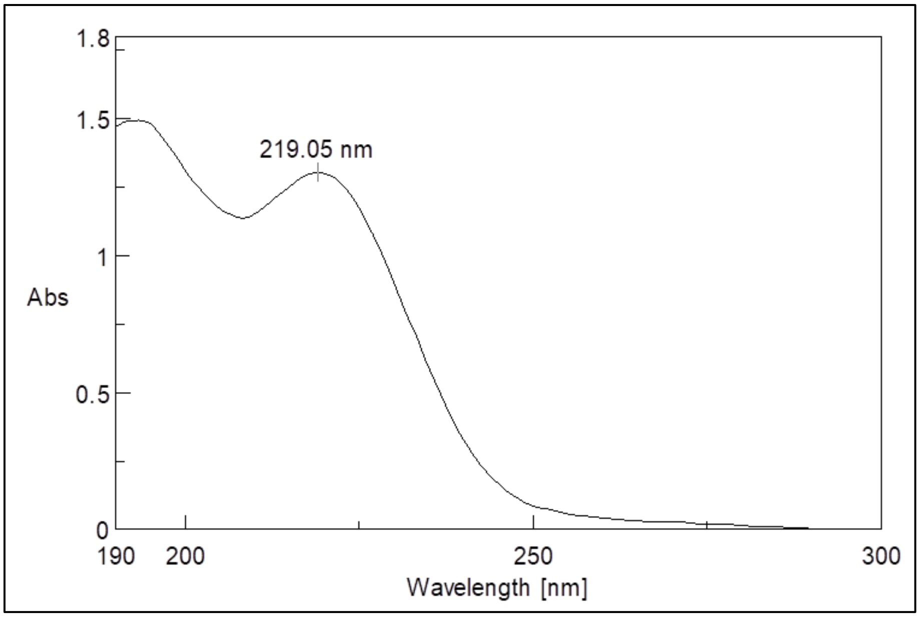

Figure S18 UV spectrum of **3** (0.05 mg/ml, dissolved in acetonitrile)

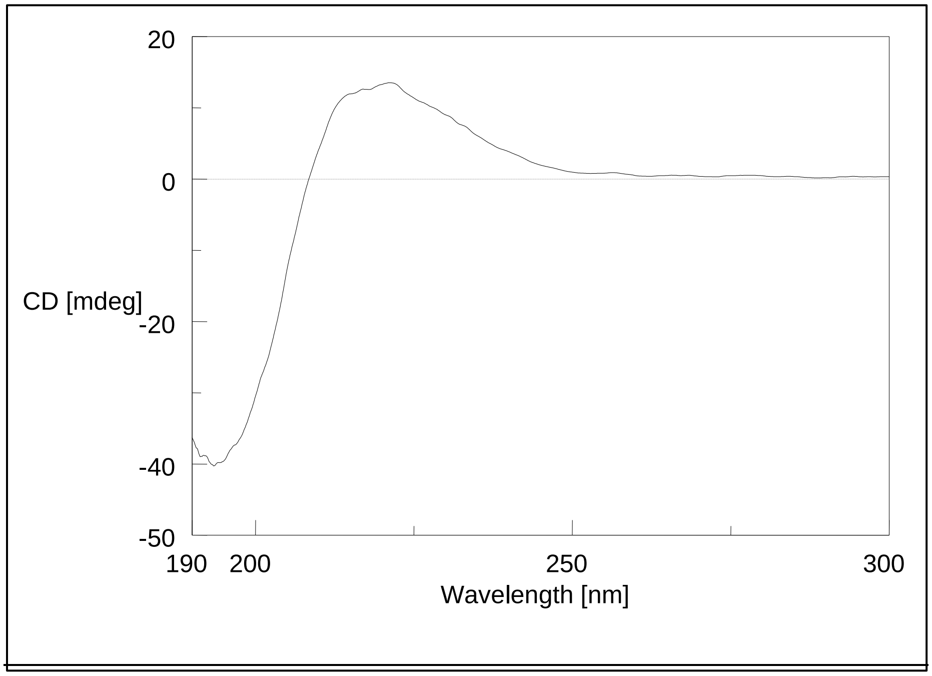

Figure S19 ECD spectrum of **3** (0.05 mg/ml, dissolved in acetonitrile)

Identification of 8-hydroxy-labd-13-en-15-oic acid (4)

Table S4 NMR data of **4** (in $CDCl_3$) recorded at 400 (^1H) and 100 (^{13}C) MHz and that of Labd-13-en-8-ol-15-oic acid (in $CDCl_3$) from reported data at 250 (^1H), and 62.9 (^{13}C) MHz

Position	Labd-13-en-8-ol-15-oic acid [3]		Compound 4		
	δ_C (ppm)	δ_H (ppm) mult (*J* in Hz)	δ_C (ppm)[a],	δ_H (ppm)[a], mult·(*J* in Hz)	Main HMBC Correlations
**1**	39.8	1.61, *m*	39.9	0.94, *m*/ 1.65, *m*	
**2**	Missing	1.62, *m*	18.6	1.45, *m*/ 1.58, *m*	
**3**	41.9	1.17,*m* / 1.24, *m*	42.1	1.18, *m*/ 1.37,*m*	
**4**	Missing	-	33.4	-	
**5**	56.1	0.89, *m*	56.3	0.91, *m*	C-4, 6, 10, 20
**6**	23.5	2.25, *m*	20.7*	1.28, *m*/ 1.63, *m*	C-5, 7,8
**7**	44.7	1.35, *m*/ 1.82, *m*	44.9	1.40, *m*/ 1.89, *dt (12.0, 3,1)*	C-5, 6, 8, 9, 17
**8**	74.4	-	74.5	-	
**9**	61.3	1.06, *m*	61.5	1.06, *td (4.0)*	C-5, 7, 8, 10, 11, 12, 20
**10**	39.2	-	39.4	-	
**11**	20.5	1.62, *m*	23.7*	1.42, *m*/ 1.62, *m*	C-8, 9
**12**	44.5	2.30, *m*	44.7	2.31, *m*/ 2.24 *m*	C-11, 13, 14, 16
**13**	163.9	-	164.3	-	
**14**	114.7	5.70, *br s*	114.5	5.71, *br s*	C-12, 13, 15, 16
**15**	171.5	-	171.2	-	
**16**	19.4	2.17, *s*	19.6	2.18, *d (1.2)*	C-12, 13, 14
**17**	24.0	1.15, *s*	24.2	1.16, *s*	C-6, 7, 8, 9
**18**	21.5	0.78, *s*	21.6	0.79, *s*	C-3, 5, 4, 19
**19**	33.4	0.86, *s*	33.5	0.87, *s*	C-5, 3, 4, 18
**20**	15.4	0.79, *s*	15.6	0.80, *s*	C-1, 5, 9, 10

[a]Assignments were based on ^1H, ^{13}C, and HSQC experiments

*The NMR chemical shifts at positions 6 and 11 were reversed compared to the reference, their accuracy was confirmed using 2D-NMR techniques, including HSQC and HMBC

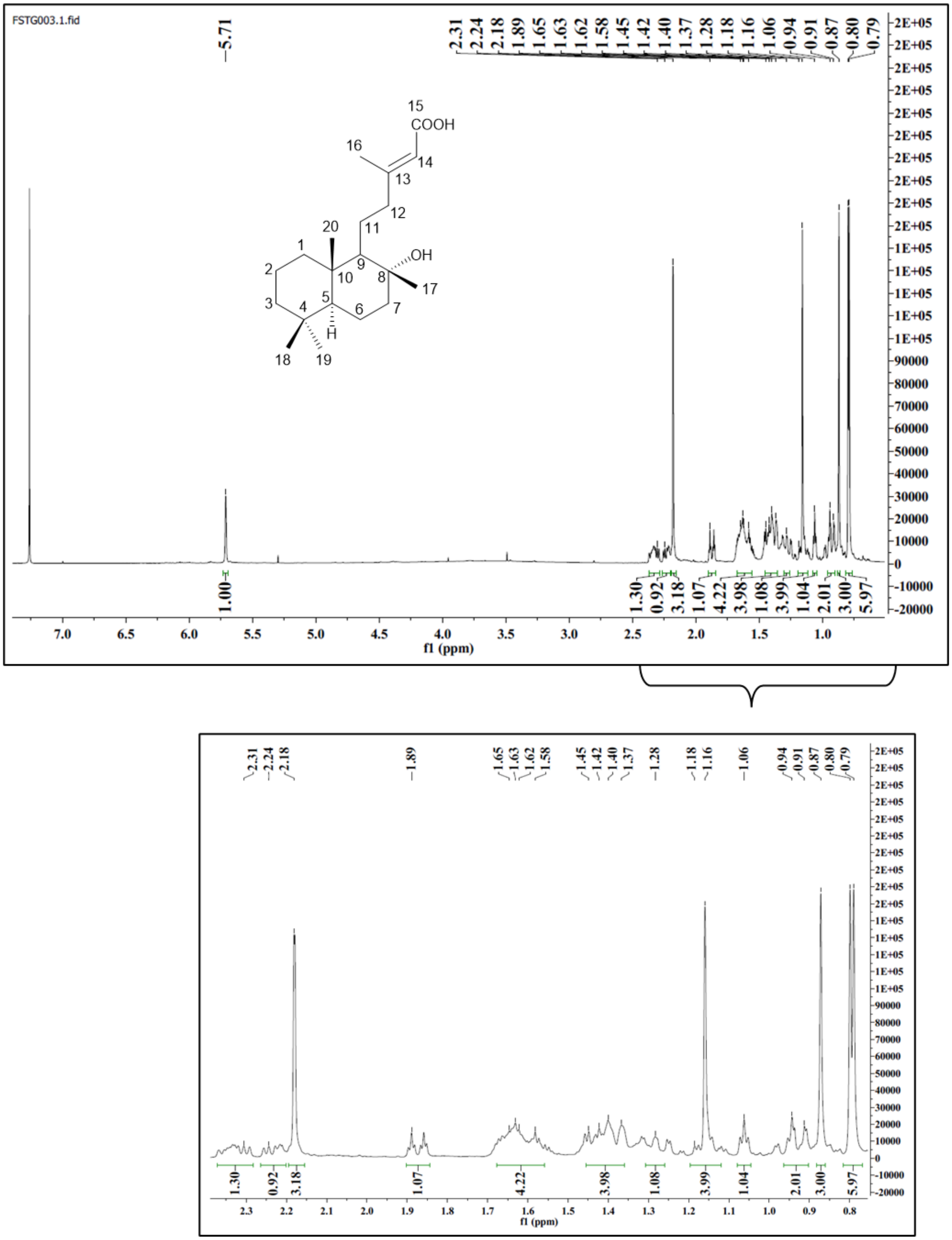

Figure S20 ^{1}H−NMR spectrum of **4** (400 MHz, $CDCl_3$)

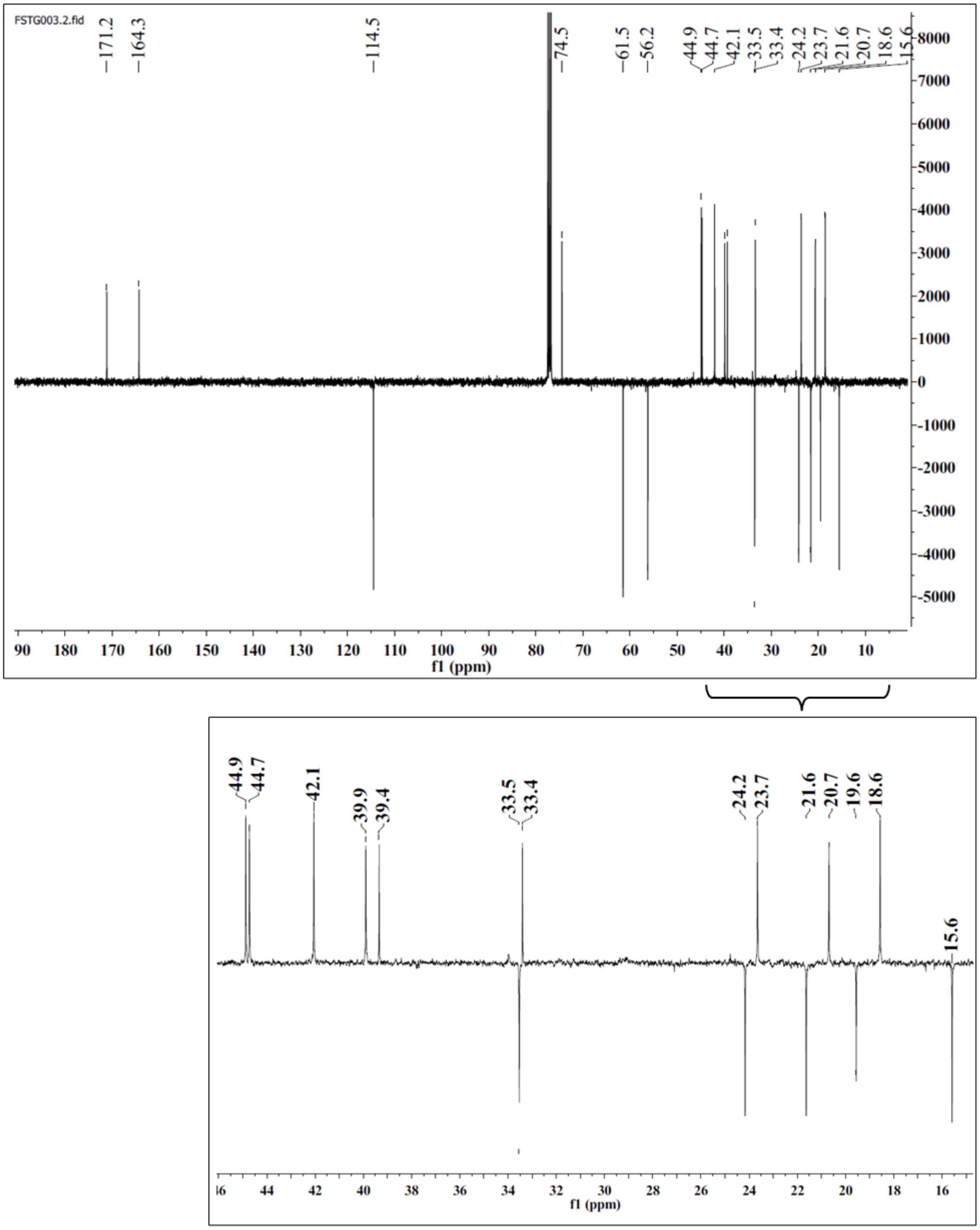

Figure S21 ^{13}C−NMR spectrum of **4** (DEPT-Q, 100 MHz, $CDCl_3$)

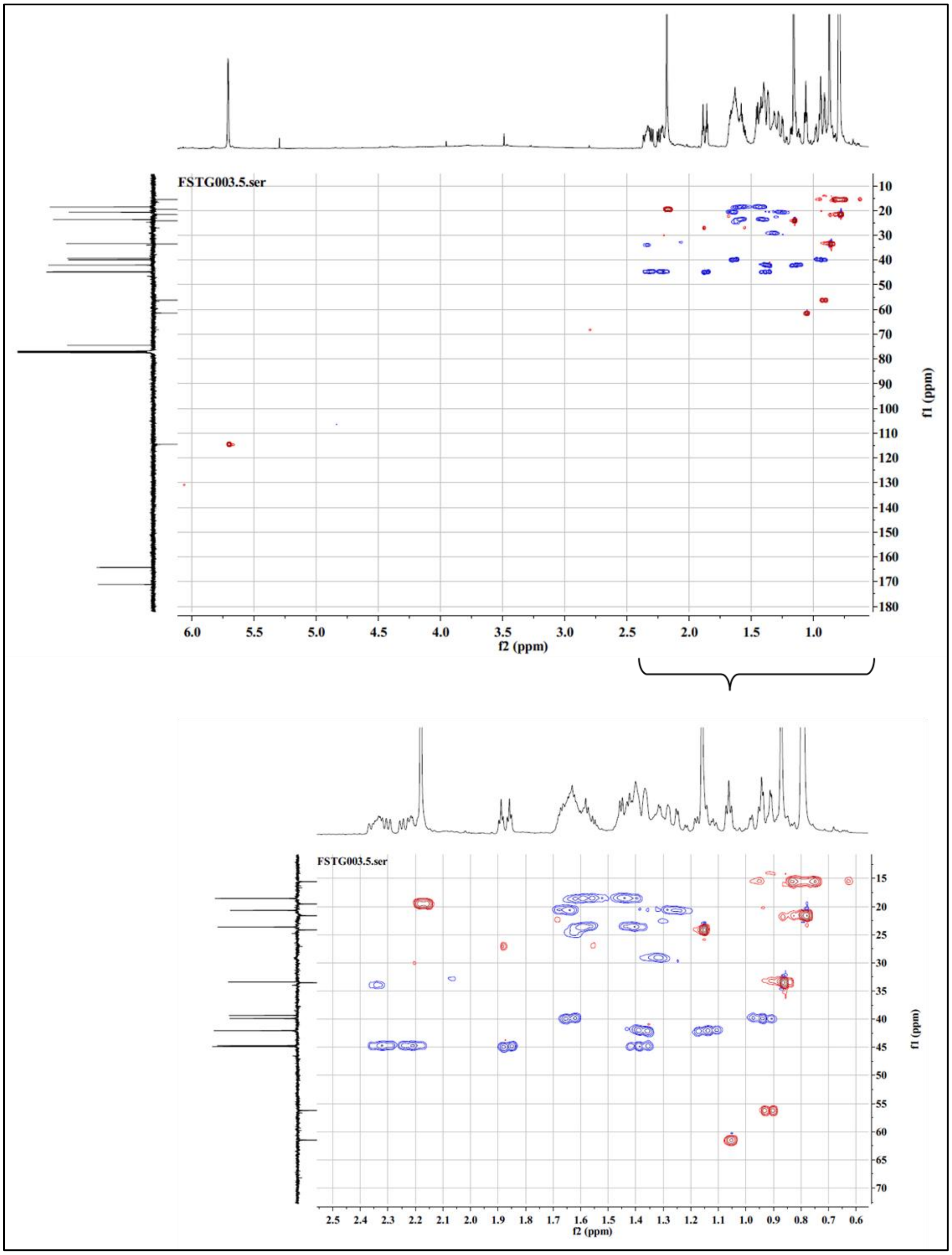

Figure S22 HSQC spectrum of **4** (400 MHz for ^{1}H and 100 MHz, for ^{13}C, $CDCl_3$)

(Blue spot; C, CH_2/Red spot; CH, CH_3)

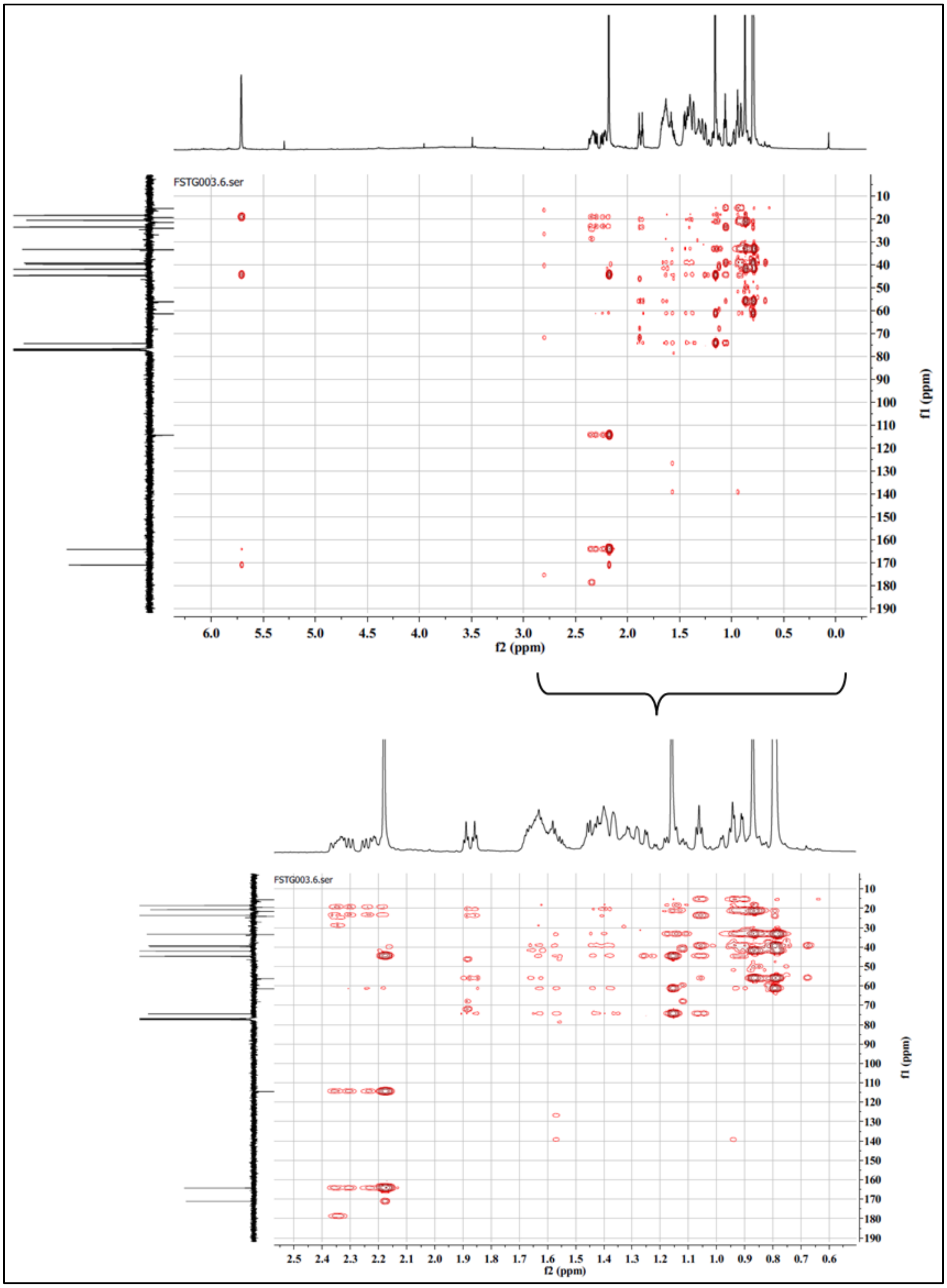

Figure S23 HMBC spectrum of **4** (400 MHz for ^{1}H and 100 MHz, for ^{13}C, $CDCl_3$)

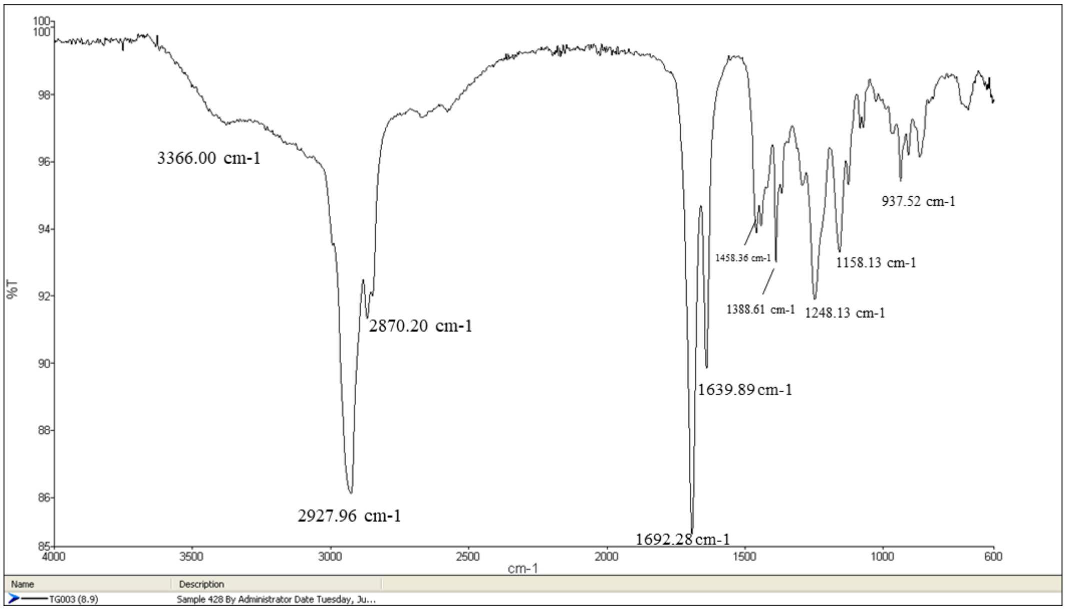

Figure S24 IR spectrum of **4**

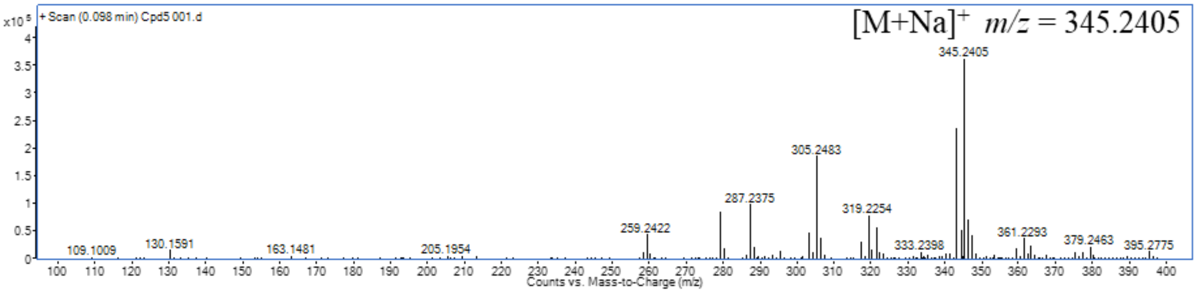

Figure S25 HRESI−MS (positive ion mode) spectrum of **4**

The MS information of the features identified by the MS-MS technique

Table S5 MS-MS fragmentation of 10 features

Feature	m/z	adduct	MS-MS fragmentation
1	337.2391	$[M+H]^+$	321.2376,303.2274,285.2172,205.1558
2	317.2138	$[M-H]^-$	273.2231,219.1749,149.0968,98.0378
3	321.2404	$[M+H]^+$	303.2276,275.2330,109.0998,69.0690
4	303.2303	$[M+H-H_2O]^+$	257.2218,163.1454,109.0995,69.0691
5	305.2445	$[M+H-H_2O]^+$	249.1802,221.1501,163.1452,109.0992,83.0843
7	323.2601	$[M-H]^-$	263.2391,183.0128,127.1130,57.0352
8	321.2449	$[M+H-H_2O]^+$	249.1798,163.1449,149.1299
9	303.2340	$[M-H]^-$	99.0459
10	305.2498	$[M-H]^-$	249.1462,163.0013
11	303.2341	$[M-H]^-$	98.0361,59.9841

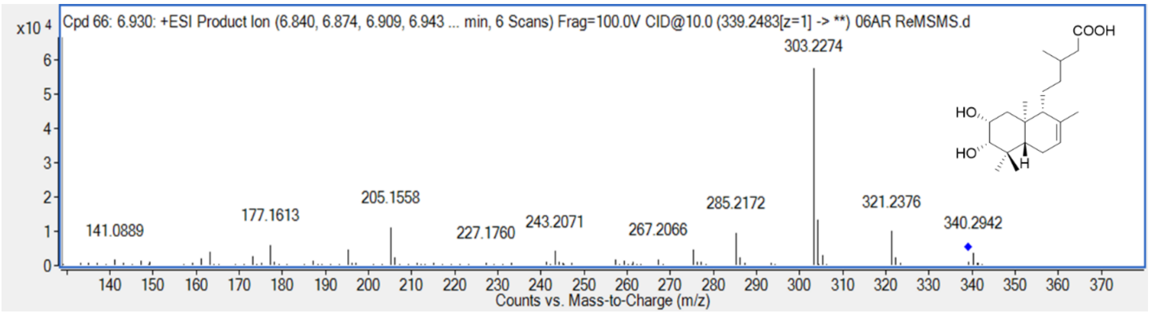

Figure S26 MS-MS fragmentation (positive ion mode) of feature 1

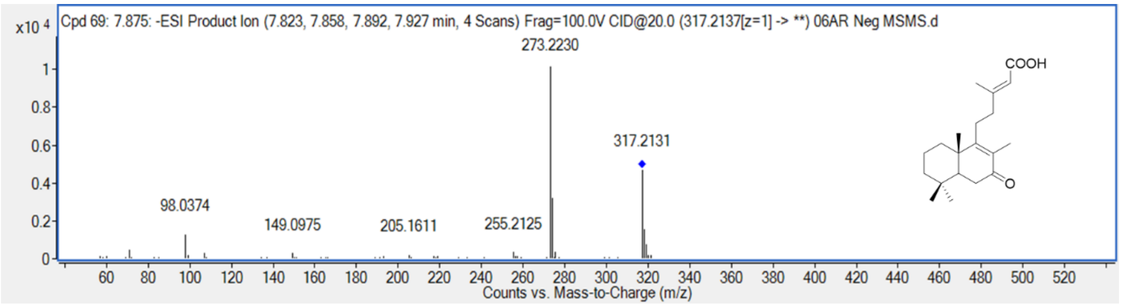

Figure S27 MS-MS fragmentation (negative ion mode) of feature 2

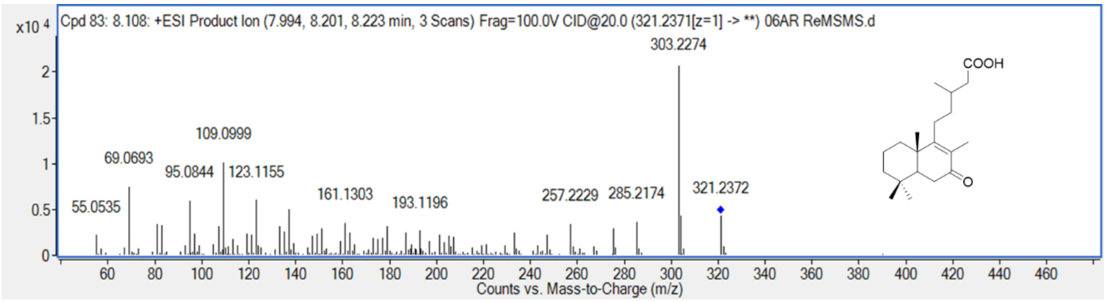

Figure S28 MS-MS fragmentation (positive ion mode) of feature 3

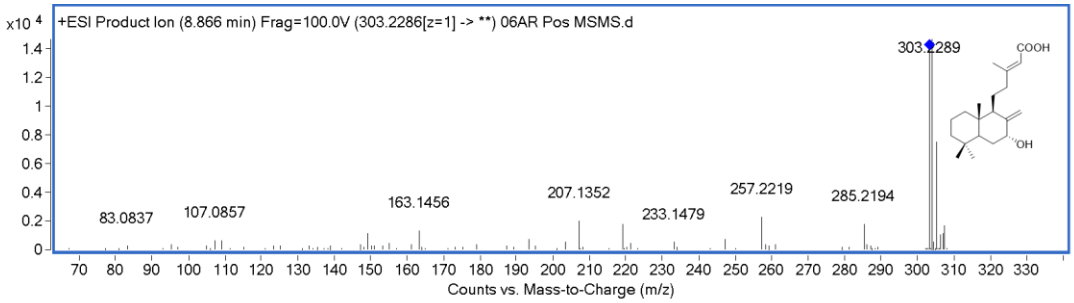

Figure S29 MS-MS fragmentation (positive ion mode) of feature 4

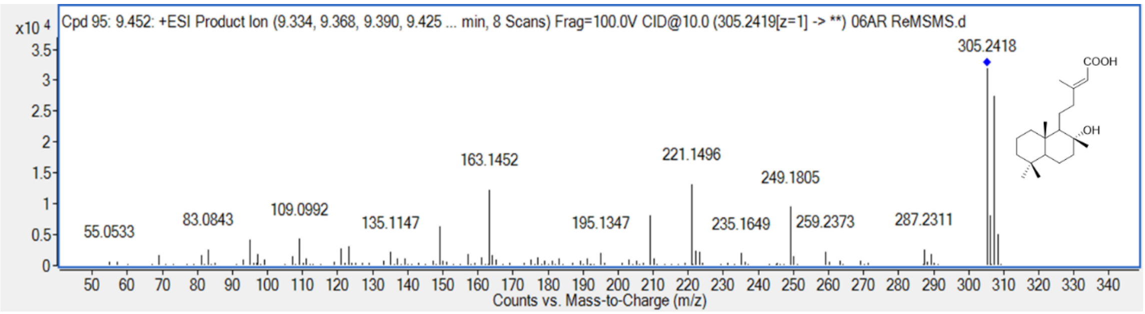

Figure S30 MS-MS fragmentation (positive ion mode) of feature 5

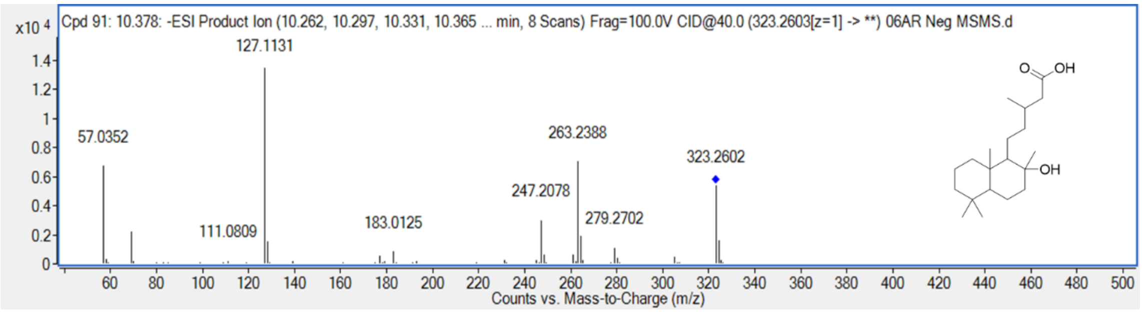

Figure S31 MS-MS fragmentation (negative ion mode) of feature 7

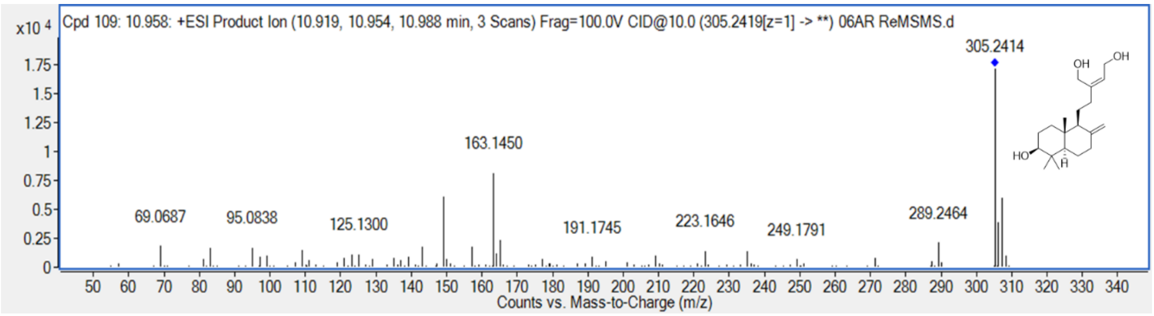

Figure S32 MS-MS fragmentation (positive ion mode; [M+H-H2O]$^{+}$) of feature 8

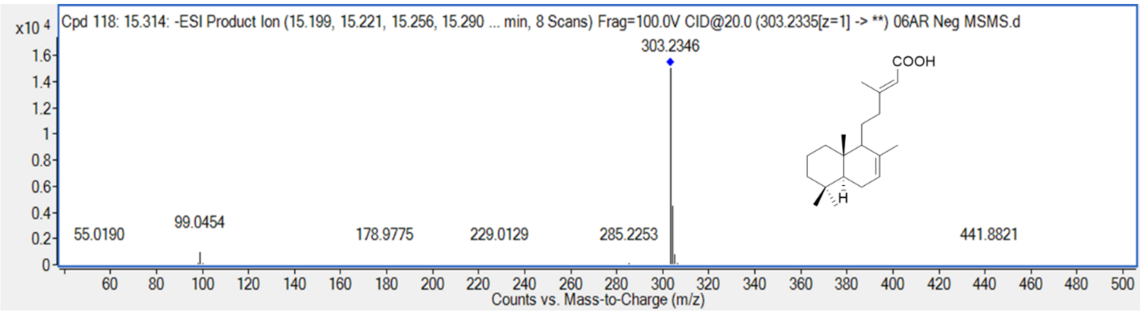

Figure S33 MS-MS fragmentation (negative ion mode) of feature 9

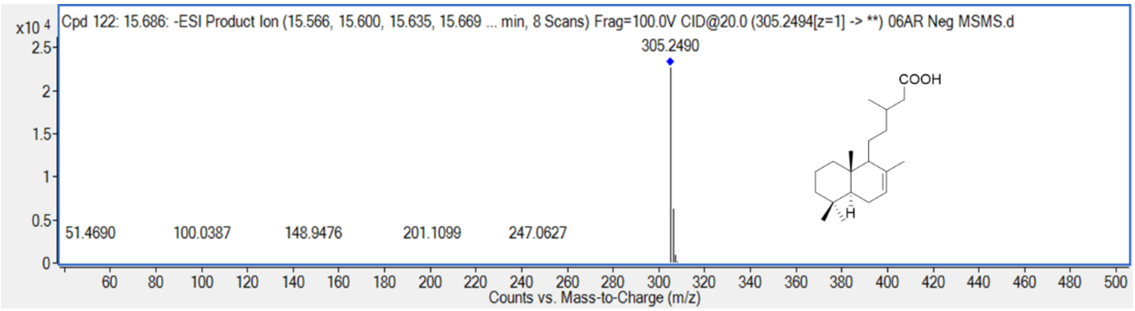

Figure S34 MS-MS fragmentation (negative ion mode) of feature 10

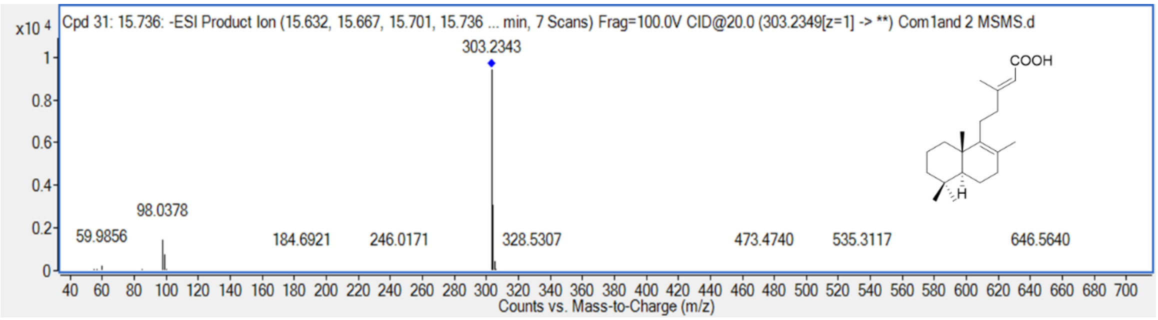

Figure S35 MS-MS fragmentation (negative ion mode) of feature 11

References

1. Dekker, T.; Fourie, T.; Matthee, E.; Snyckers, F.; Van der Schyf, C.; Boeyens, J.; Denner, L. Studies of South African medicinal plants: Pt. 7. *South African Journal of Chemistry* **1988**, *41* (1), 33-35

2. Rijo, P. Phytochemical study and biological activities of diterpenes and derivatives from *Plectranthus* species. Ph.D. Thesis, universidade de lisboa, Portugal, **2011**

3. Baratta, M.; Ruberto, G.; Tringali, C. Constituents of the pods of Piliostigma thonningii. *Fitoterapia (Milano)* **1999**, *70* (2), 205-208.
